# Oncolytic vaccinia virus injected intravenously sensitizes pancreatic neuroendocrine tumors and metastases to immune checkpoint blockade

**DOI:** 10.1016/j.omto.2021.12.016

**Published:** 2021-12-21

**Authors:** Mitsuko Inoue, Minah Kim, Tomoyoshi Inoue, Madeline Tait, Thomas Byrne, Maximilian Nitschké, Patrizia Murer, Howard Cha, Aishwarya Subramanian, Naomi De Silva, Teresa Chiaverotti, Donald M. McDonald

**Affiliations:** 1 UCSF Helen Diller Family Comprehensive Cancer Center, Cardiovascular Research Institute and Department of Anatomy, University of California, San Francisco, 513 Parnassus Avenue, Room S1349, San Francisco, CA 94143-0452, USA; 2 SillaJen Biotherapeutics Inc., San Francisco, CA 94111, USA

**Keywords:** apoptosis, cytotoxic T cells, NK cells, insulin, hypoglycemia, liver metastasis, regression, PD-1, Pexa-Vec, RIP1-Tag2 mice, A/J mice

## Abstract

This study determined the influence of intravenous (i.v.) oncolytic vaccinia virus mpJX-594 (mpJX) on antitumor activity of anti-programmed death receptor-1 antibody (aPD1) in functional and metastatic pancreatic neuroendocrine tumors (PanNETs). One i.v. dose of mpJX, engineered for mice with the same plasmid design as clinical virus Pexa-Vec, was administered alone or with repeated dosing of aPD1 (mpJX+aPD1) to two contrasting genetic models of PanNET: one developing benign insulin-secreting tumors (RIP1-Tag2;C57BL/6J mice) and the other developing liver metastases (RIP1-Tag2;AB6F1 mice). Experiments revealed that aPD1 had synergistic actions with mpJX on CD8^+^ T cell and natural killer (NK) cell influx, apoptosis, and suppression of proliferation in PanNETs. After mpJX+aPD1, the 53-fold increase in apoptosis (5 days) and 85% reduction in proliferation (20 days) exceeded the sum of mpJX and aPD1 given separately. mpJX+aPD1 also stabilized blood insulin and glucose in mice with functional PanNETs, regressed liver metastases in mice with aggressive PanNETs, and prolonged survival of both. The findings revealed that mpJX+aPD1 converted “cold” PanNETs into immunogenic tumors with widespread cytotoxic T cell influx, tumor cell killing, and suppression of proliferation. Reduction of tumor insulin secretion from functional PanNETs prolonged survival, and anti-metastatic actions on aggressive PanNETs reduced the metastatic burden to less than before treatment. The findings support the efficacy of the vaccinia virus with aPD1 for functional and metastatic PanNETs.

## Graphical abstract

**Figure:**
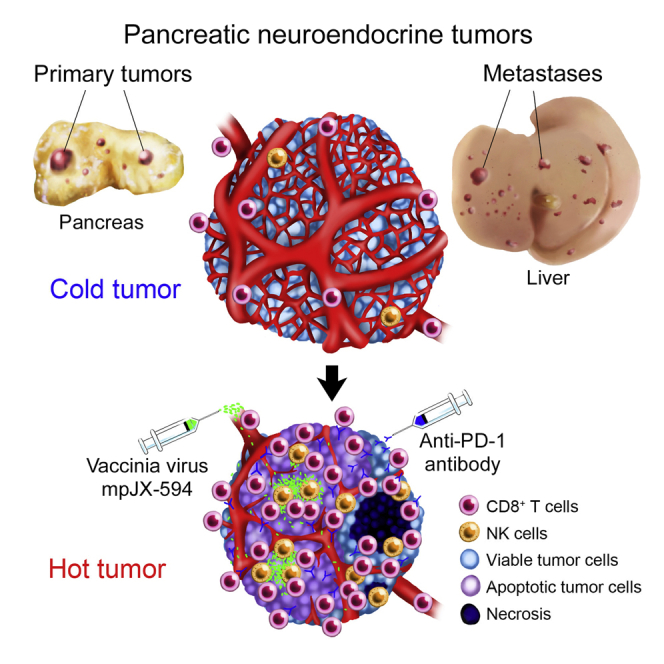

## Teaser

Vaccinia virus mpJX-594 administered intravenously to mice with pancreatic neuroendocrine tumors increases the antitumor response of an anti-PD1 antibody by turning immunologically “cold” tumors into “hot” tumors. Synergistic actions of the treatment combination amplify cytotoxic T cell influx and lead to smaller, less functional primary tumors and to the regression of metastases.

## Introduction

Immune checkpoint blockade, which promotes antitumor immunity by targeting programmed death receptor-1(PD-1), PD-ligand 1 (PD-L1), or other immune checkpoints has promising efficacy in some cancers. Numerous studies have reported clinical responses to immune checkpoint inhibition in a subset of patients with immunogenic tumors with cytotoxic T cell infiltration.^1^

Neuroendocrine neoplasms (NENs), classified by the World Health Organization as pancreatic neuroendocrine tumors (PanNETs), poorly differentiated pancreatic neuroendocrine carcinomas (PanNECs), and other advanced NENs,^2^ are among the cancers in which immune checkpoint inhibitors have been used.^3^^,^^4^ However, limited efficacy in these patients^5^ drives the search for combinations with other agents that increase responses by turning immunologically “cold” tumors into “hot” tumors.^1^

Oncolytic viruses that amplify antitumor responses are among the treatment combinations being assessed for increasing susceptibility to immune checkpoint blockade.^6^, ^7^, ^8^, ^9^, ^10^, ^11^, ^12^ The approach is supported by preclinical and clinical studies showing that oncolytic viruses increase cytotoxic and memory T lymphocyte influx and promote antitumor immunity.^13^^,^^14^

Pexa-Vec (JX-594, pexastimogene devacirepvec) is a Wyeth strain oncolytic vaccinia virus engineered with viral thymidine kinase gene disruption and human granulocyte-macrophage colony-stimulating factor (hGM-CSF) transgene expression to favor replication selectivity for tumor cells and promote immune-activation.^6^^,^^7^^,^^15^ Antitumor activity and tolerability have been documented in preclinical and clinical studies.^6^^,^^7^^,^^15^^,^^16^ Pexa-Vec administered by intravenous (i.v.) or intratumoral (i.t.) injection in combination with immune checkpoint inhibitors is now being examined in clinical trials of metastatic or unresectable renal cell carcinomas and other solid tumors (ClinicalTrials.gov: NCT03294083 and NCT03206073).

Vaccinia virus mpJX-594 (mpJX), engineered from the mouse-adapted Western Reserve (WR) strain using the same plasmid design as was used for Pexa-Vec, infects tumor vasculature and tumor cells after i.v. administration and triggers a robust immune response in RIP1-Tag2 (RT2) transgenic mice that develop PanNETs.^16^ The infection and immune response are accompanied by widespread tumor cell killing that continues for at least 10 days after one i.v. dose of mpJX and 30 days after two doses.^16^ Viral GM-CSF appears to play a minor role at 5 days in those studies because apoptosis increased similarly regardless of viral expression of hGM-CSF, mouse GM-CSF (mGM-CSF), or no GM-CSF.^16^

Another engineered WR strain variant of Pexa-Vec, which expresses mGM-CSF, administered by i.t. injection in combination with anti-PD-1 antibody (aPD1), is reported to promote greater CD4^+^ and CD8^+^ T cell infiltration, tumor cell killing, and growth slowing of subcutaneous renal cell carcinomas (Rencas) in syngeneic mice and mammary tumors in MMTV-PyMT mice.^10^ These findings further support the rationale for combining vaccinia viruses with aPD1; however, viral injection into individual tumors was required because i.v. administration appeared ineffective.^10^ This brought into question the potential for using i.v. administration of vaccinia viruses to infect and promote tumor immunity and killing in primary tumors and widespread metastases.

With this background, we determined whether i.v. administration of mpJX can amplify the antitumor activity of PD-1 blockade on spontaneous PanNETs in transgenic mice that develop immunologically cold tumors responsive to the vaccinia viruses.^16^^,^^17^ To explore mechanisms underlying the greater antitumor activity of aPD1 when given together with mpJX (mpJX+aPD1), we asked whether the combination has synergistic activity on viral infection, immune cell influx, tumor cell killing, and growth suppression. As PanNETs in transgenic mouse models have two contrasting phenotypes, functional and relatively benign PanNETs in RT2;C57BL/6J (RT2;B6) mice^18^^,^^19^ and poorly functional, highly metastatic PanNETs in hybrid RT2;AB6F1 (RT2;AB6F1) mice,^19^, ^20^, ^21^ we used the former to determine the treatment efficacy on reducing insulin secretion from functional PanNETs and used the latter to test the regression of liver metastases from aggressive PanNETs.

Experiments revealed that one i.v. dose of mpJX with repeated dosing of aPD1 had synergistic activity in promoting immune cell influx, increasing tumor cell killing, and suppressing proliferation. Hypoglycemia was ameliorated in mice with functional PanNETs, metastatic tumor burden was reduced in mice with aggressive PanNETs, and survival was prolonged in both.

## Results

The influence of i.v. administration of the vaccinia virus mpJX with concurrent checkpoint blockade by aPD1 was assessed by determining (1) the balance of tumor cell killing and growth suppression in spontaneous PanNETs in RT2;B6 mice; (2) the time course of infection, types of immune cells recruited, and amount of vascular remodeling in tumors in RT2;B6 mice as factors contributing to antitumor activity; and (3) the effects on liver metastasis in RT2;AB6F1 mice.

### Balance of tumor cell killing and growth suppression

**Figure 1:**
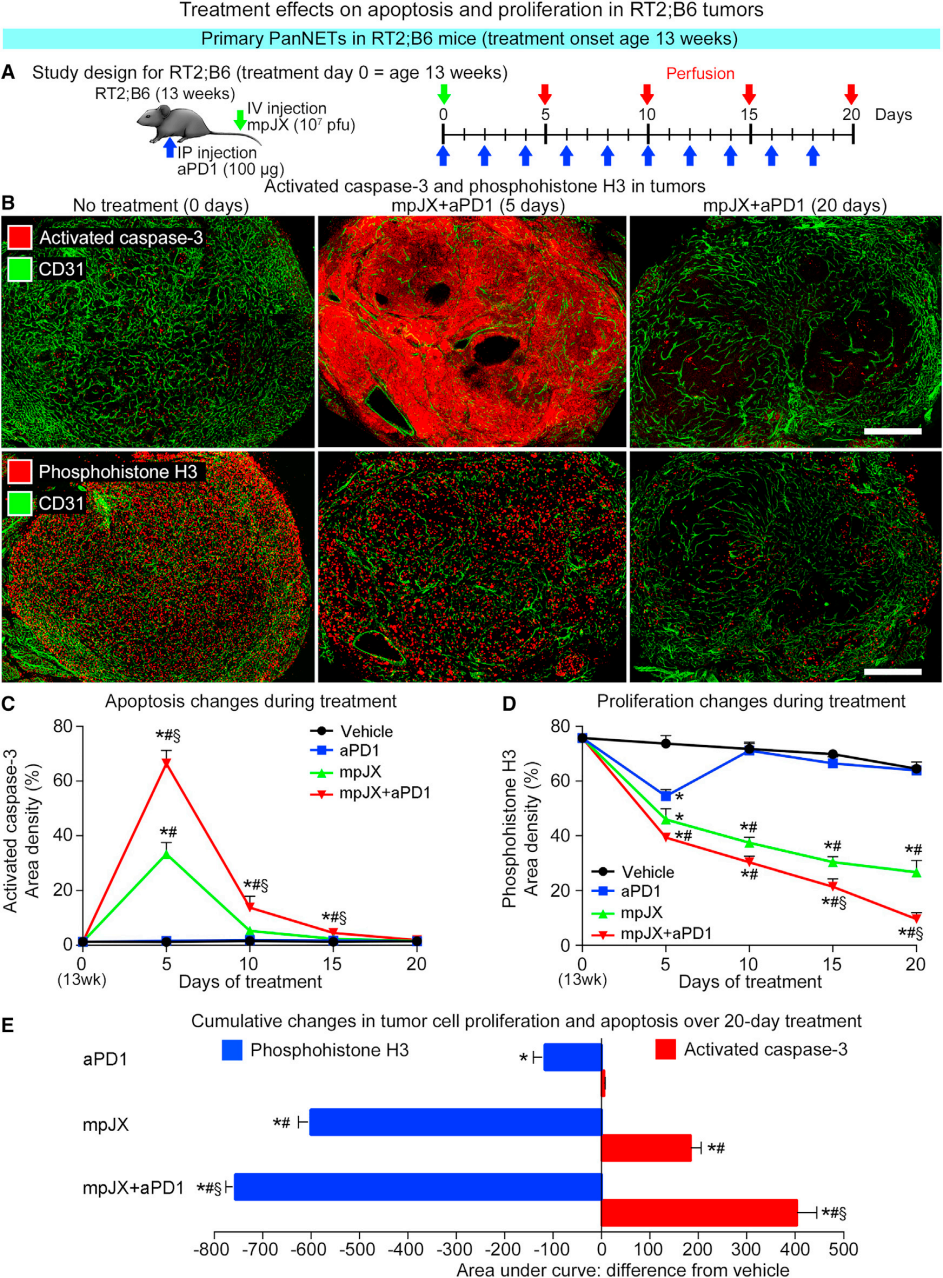
Study design and temporal changes in apoptosis and proliferation in primary PanNETs in RT2;B6 mice after treatment (A) Design of studies of 13-week-old RT2;B6 mice that received one i.v. dose of mpJX (10^7^ PFU) or Vehicle (PBS) on day 0 (green arrow) and aPD1 (100 μg) or normal rat IgG2a injected i.p. every other day (blue arrows). Mice were perfused with fixative 5, 10, 15, or 20 days after treatment onset (red arrows). (B) Confocal microscopic images of staining for activated caspase-3 (red, top row) and phosphohistone H3 (red, bottom row) and blood vessels (CD31, green) on days 0, 5, and 20 after mpJX+aPD1. Scale bar, 500 μm. (C) Line plots of mean area density of activated caspase-3 in 5 largest tumors of all mice in each group. Values changed little after Vehicle or aPD1 throughout the 20-day experiment but increased rapidly to a peak 5 days after mpJX (33% area density) and were twice as widespread after mpJX+aPD1 (66% area density). Values for all treatments returned to the low baseline level at 20 days. (D) Line plots of phosphohistone H3 staining in tumors showing the time-dependent reduction over 20 days after mpJX (59% reduction) and an even greater reduction after mpJX+aPD1 (85% reduction). (E) Areas under the curves in (C) and (D) showing the cumulative decreases in tumor cell proliferation and increases in apoptosis over 20 days normalized to the value for Vehicle (area = 0). The decrease in tumor cell proliferation and the increase in apoptosis after mpJX+aPD1 were significantly greater than after mpJX. (C–E) ANOVA: p < 0.05 compared with ∗Vehicle, ^#^aPD1, or ^§^mpJX. n = 5 mice/group (both sexes).

Antitumor effects of mpJX administered i.v. alone were compared with those of the virus given in combination with aPD1 (mpJX+aPD1) by measuring tumor cell killing (activated caspase-3 staining^16^) and proliferation (phosphohistone H3 staining^22^) in RT2;B6 mice 5 to 20 days after treatment onset at age 13 weeks (Figure 1A). Apoptosis was sparse in untreated tumors but was extensive after mpJX and even more widespread after mpJX+aPD1 (Figures 1B and S1A). Apoptosis peaked at 5 days (1% area density at baseline, 33% after mpJX, and 66% after mpJX+aPD1) and then diminished but was still 25% above baseline 20 days after mpJX+aPD1 (Figures 1C and S1B).

Tumor cell proliferation was widespread at baseline but was 38% less at 5 days after mpJX and 47% less after mpJX+aPD1 (Figures 1B, 1D, and S1A). Strikingly, proliferation continued to decrease and at 20 days was reduced 59% after mpJX and 85% after mpJX+aPD1 (Figures 1B, 1D, and S1B). aPD1 alone had little effect on proliferation (1% reduction).

The area under the time course curves of activated caspase-3 and phosphohistone H3 staining (Figures 1C and 1D) enabled the comparison of treatment effects on apoptosis and proliferation (Figure 1E). During the 20-day study, compared with Vehicle, apoptosis was 7.5-fold greater after mpJX and 15-fold greater after mpJX+aPD1 (Figure 1E), and proliferation was 42% less after mpJX and 53% less after mpJX+aPD1. Changes after mpJX+aPD1 were significantly greater than either mpJX or aPD1 alone (Figure 1E).

### Balance of necrotic and viable tissue to tumor size

**Figure 2:**
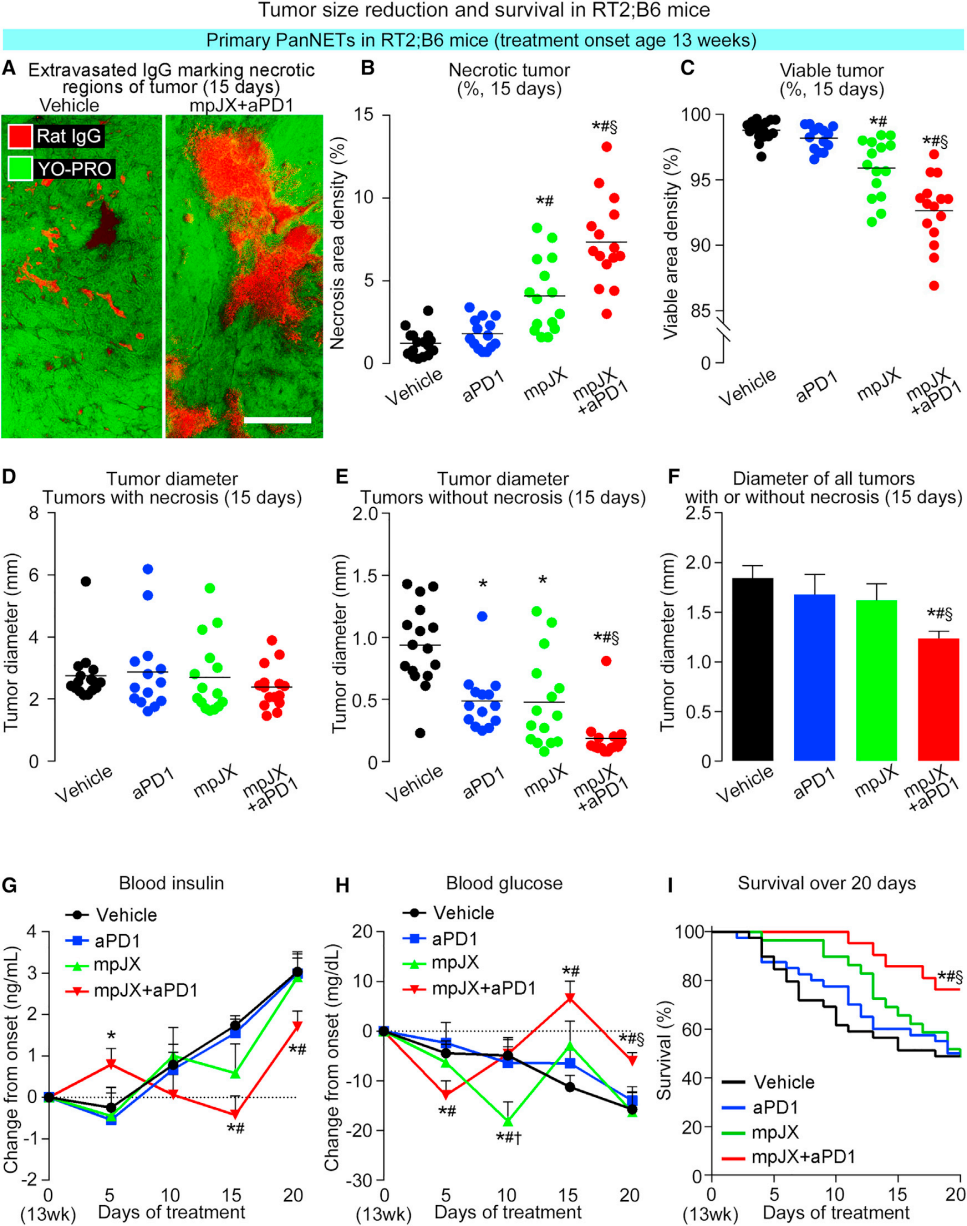
Treatment effects on necrotic and viable tumors, blood insulin and glucose, and survival of RT2;B6 mice (A) Fluorescence microscopic images of tumors 15 days after Vehicle (left) or mpJX+aPD1 (right) comparing amount and distribution of necrosis marked by extravasated rat IgG (Cy3 anti-rat IgG, red) and the absence of cell nuclei (YO-PRO-1, green). Scale bar, 400 μm. (B–D) Area density (percentage of tumor area) of necrosis and viable tumor (total area minus necrosis) and diameter of 5 largest tumors (diameter >1.5 mm), where dots represent mean value for each mouse. Proportion of necrosis was significantly greater and viable tumor was significantly less 15 days after mpJX+aPD1 than after all other treatments, but the mean diameters were not significantly different. ANOVA: p < 0.05 compared with ∗Vehicle, ^#^aPD1, or ^§^mpJX. n = 14–16 mice/group. (E and F) Significant reduction in mean diameter of next largest 5 tumors (diameter <1.5 mm), which had little or no necrosis, and of all 10 tumors, necrotic area excluded, after mpJX+aPD1 but not after other treatments. Student’s t test: p < 0.05 compared with ∗Vehicle, ^#^aPD1, or ^§^mpJX. n = 14–16 mice/group. (G) Blood insulin (change from mean at onset, 3.9 ng/mL) increased from the onset age of 13 weeks to the age of 16 weeks in all groups except mpJX+aPD1, where values were lower at ages 15 and 16 weeks (treatment days 15 and 20). (H) Blood glucose (change from mean onset value of 40 mg/dL) decreased over time in all groups except mpJX+aPD1, where values were higher at ages 15 and 16 weeks. Student’s t test: p < 0.05 compared with Vehicle∗, aPD1^#^, or mpJX^§^. n = 11–23 mice/group. (I) Kaplan-Meier plots showing greater survival of mice until end of experiment at age 16 weeks (13 weeks + 20 days) after mpJX+aPD1 (76.2% of 21 mice) than after Vehicle (48.7% of 39 mice), aPD1 (50.0% of 40 mice), or mpJX (48.3% of 29 mice). Log-rank test: p < 0.05 compared with ∗Vehicle, ^#^aPD1, or ^§^mpJX.

The consequences of treatment-related apoptosis and proliferation were assessed by measuring overall tumor size and the amounts of necrotic and viable tumor in RT2;B6 mice after 15 days of treatment. Necrosis was identified by the absence of DAPI/YO-PRO-1 staining of nuclei and by the non-specific accumulation of extravasated aPD1 or immunoglobulin G (IgG)2a (Figures 2A, S1C, and S1D). Non-necrotic regions were considered viable. As the 5 largest tumors (diameter >1.5 mm) had much more necrosis than the next 5 largest tumors (diameter <1.5 mm), the two groups were evaluated separately. In the 5 largest tumors, the proportion of sectional areas of necrosis was 2.4 times larger after mpJX and 5 times larger after mpJX+aPD1 (Figure 2B). Accordingly, the proportion of viable tumor was significantly less after these treatments (Figure 2C), but overall tumor size had little change (Figures 2D and S2A). By comparison, the next 5 largest tumors, which had little or no necrosis, and both groups of tumors considered together, regardless of the presence of necrosis, were smaller after mpJX+aPD1 (Figures 2E, 2F, S2B, and S2C).

### Relation of PanNET functionality to survival

As RT2;B6 mice usually die from hypoglycemia due to functional PanNET and not from tumor burden,^16^^,^^23^ we asked whether blood insulin and glucose were stabilized by treatment. In untreated RT2;B6 mice from age 10 to 16 weeks, tumor size increased 3-fold, blood insulin increased to more than 2 ng/mL, glucose decreased to less than 30 mg/dL, and mortality reached 65% (Figures S2D–S2G). By comparison, 20 days after mpJX+aPD1, insulin averaged 44% lower and glucose 61% higher (Figures 2G and 2H). These improvements were not found after aPD1 or mpJX. Stabilization of blood insulin and glucose by mpJX+aPD1 was accompanied by 56% better survival of RT2;B6 mice until euthanasia at age 16 weeks (Figure 2I).

### Relation of vaccinia infection to antitumor action

**Figure 3:**
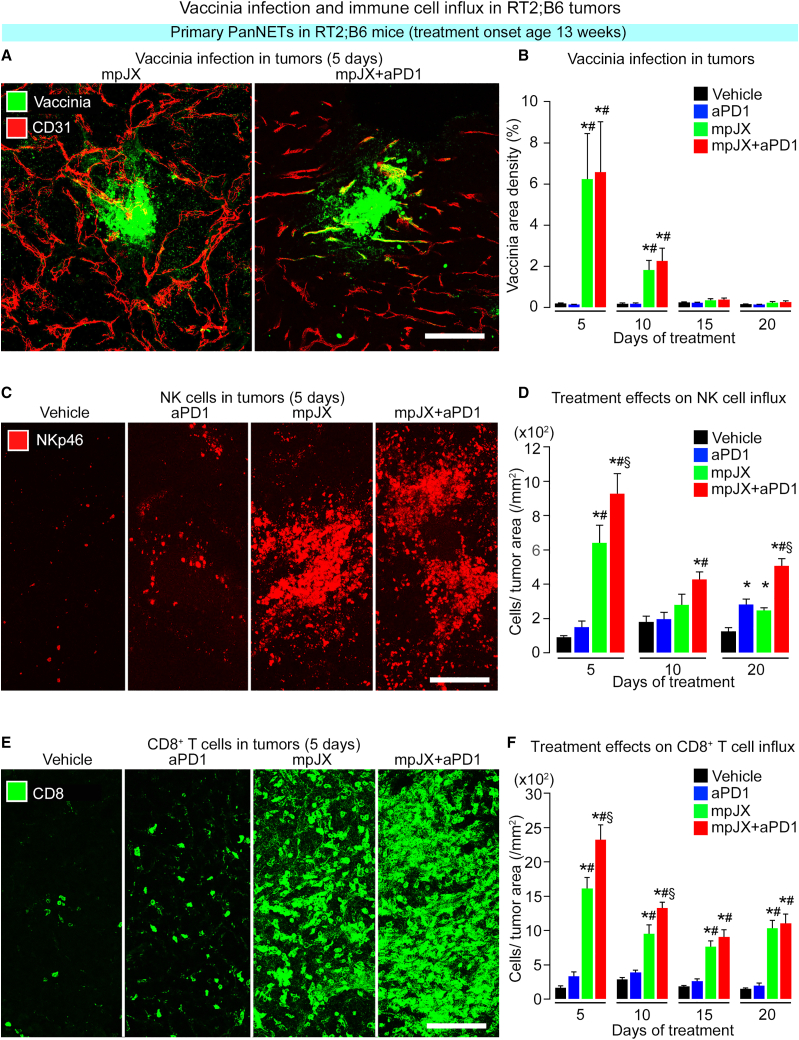
Vaccinia infection and immune cell influx in tumors of RT2;B6 mice (A) Confocal microscopic images of vaccinia (green) and tumor blood vessels (CD31, red) 5 days after mpJX (left) or mpJX+aPD1 (right). (B) Mean area density of vaccinia in 5 largest tumors in all mice in each group. Vaccinia staining was similar after mpJX (6.2%) and mpJX+aPD1 (6.6%). (C) Confocal microscopic images showing that NK cells (NKp46, red) were sparse at 5 days in the control (Vehicle), more numerous after mpJX, and even more abundant after mpJX+aPD1. (D) Measurements showing significantly more NK cells at 5, 10, and 20 days after mpJX+aPD1 than other treatments. NK cells were also more abundant at 20 days after aPD1 or mpJX but were only half as numerous as after mpJX+aPD1. (E) Confocal microscopic images showing that CD8^+^ cells (CD8, green) were sparse at 5 days in the control (Vehicle), more numerous after mpJX, and even more abundant after mpJX+aPD1. (F) Measurements showing significantly more CD8^+^ cells at 5, 10, 15, and 20 days after mpJX or mpJX+aPD1. Values 5 and 10 days after mpJX+aPD1 are greater than after mpJX. Scale bar, 100 μm in all images. (B, D, and F) ANOVA: p < 0.05 compared with ∗Vehicle, ^#^aPD1, or ^§^mpJX. n = 8–12 mice/group (both sexes).

The contribution of the vaccinia infection to widespread tumor cell killing after mpJX+aPD1 was assessed by comparing amounts of infection and apoptosis in RT2;B6 mice. Staining for vaccinia antigen was strong in tumors but restricted to focal patches 5 days after one dose of mpJX and was essentially the same after mpJX+aPD1 (Figures 3A and 3B). Vaccinia was weak or absent 10 days or longer after either treatment (Figure 3B). By comparison, apoptosis was 5 times more extensive than vaccinia 5 days after mpJX and 10 times more widespread after mpJX+aPD1 (Figures S2H and S2I).

### Recruitment of CD8^+^ T cells, natural killer (NK) cells, and other immune cells to tumors

Effects of mpJX and mpJX+aPD1 on immune cell recruitment to PanNET in RT2;B6 mice were assessed by immunohistochemistry and flow cytometry. At 5 days, NK cells, identified by NKp46^+^ immunoreactivity, were sparse in tumors after Vehicle or aPD1 but increased 6-fold after mpJX and 10-fold after mpJX+aPD1 (Figures 3C and 3D). 20 days after mpJX+aPD1, NKp46^+^ cells were fewer but still 3 times the baseline (Figures 3D and S3A).

**Figure 4:**
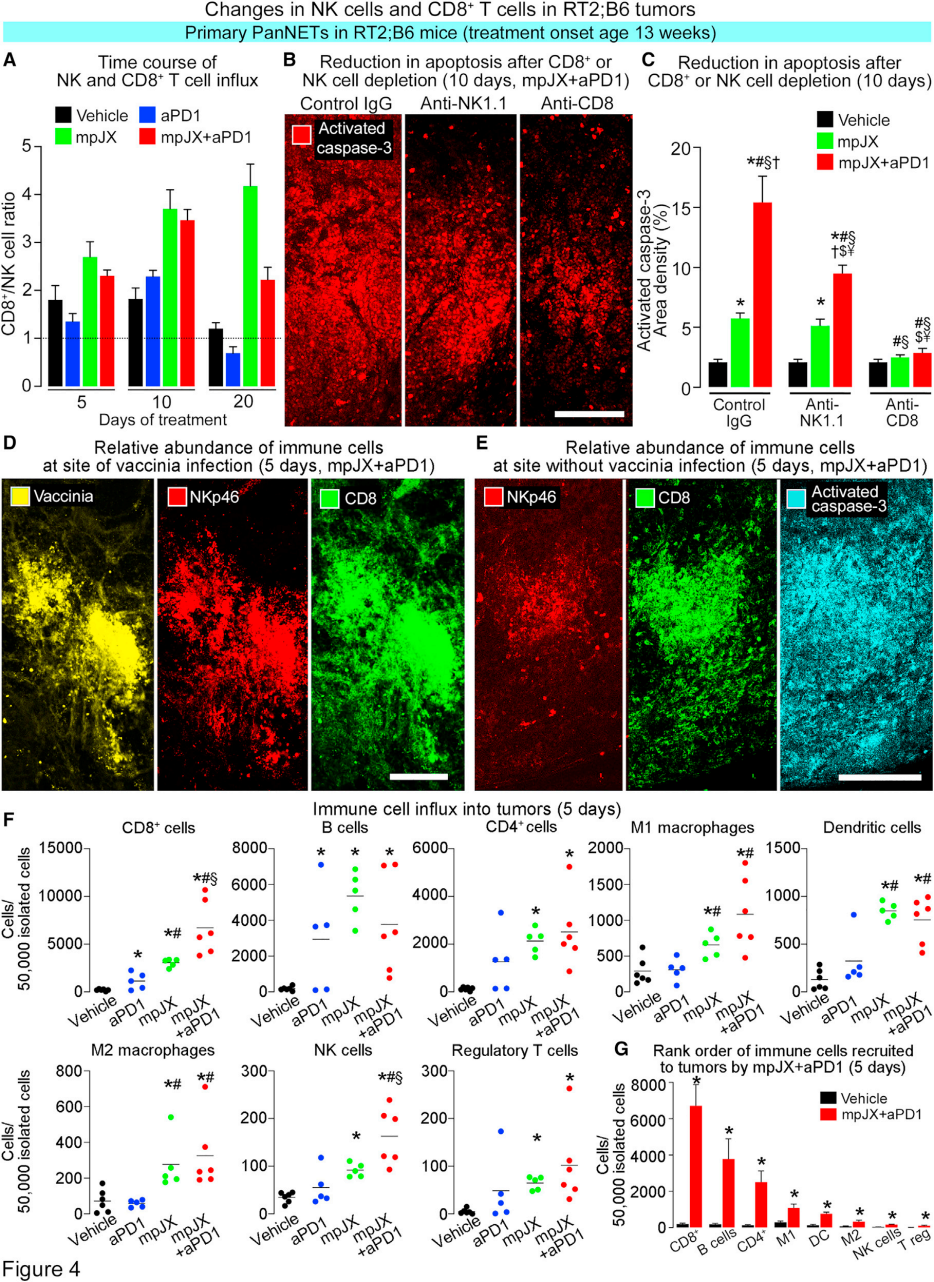
Changes in NK cells, CD8^+^ T cells, and other immune cells in tumors of RT2;B6 mice (A) CD8^+^ T cell/NK cell ratios showing the dominance of CD8^+^ cells from 5 to 20 days after mpJX or mpJX+aPD1. n = 7–12 mice/group. (B) Confocal microscopic images showing the effect of depletion of NK cells or CD8^+^ cells on apoptosis (activated caspase-3, red) in RT2;B6 tumors at 10 days. Less apoptosis is present when mpJX+aPD1 was administered after NK cell depletion and even less after CD8^+^ cell depletion. Scale bar, 100 μm. (C) Measurements showing apoptosis (activated caspase-3) in tumors when mpJX or mpJX+aPD1 was accompanied by NK cell depletion and even less after CD8^+^ cell depletion. Student’s t test: p < 0.05 compared with ∗Vehicle, ^#^mpJX, or ^§^mpJX after NK-depletion, ^†^mpJX after CD8^+^-cell depletion, ^$^mpJX+aPD1, or ^¥^mpJX+aPD1 after NK-depletion. n = 5–7 mice/group. (D) Confocal microscopic images of vaccinia infection (yellow), NK cells (NKp46, red), and CD8^+^ T cells (green) showing that both cell types were abundant in regions of vaccinia antigen staining 5 days after mpJX+aPD1. (E) Confocal microscopic images of a tumor region without vaccinia antigen staining showing sparse NKp46^+^ cells (red) but abundant CD8^+^ cells (green) coinciding with widespread apoptosis (activated caspase-3, cyan) 5 days after mpJX+aPD1. (D and F) Scale bar, 200 μm. (F and G) Flow cytometric analysis of immune cells isolated from tumors of RT2;B6 mice after treatment over 5 days. Sorting strategy set out in Table S1. Each dot is the mean value for one mouse. Values are expressed as number of cells per 50,000 isolated cells. Mean ± SEM. Student’s t test: p < 0.05 compared with corresponding value for ∗Vehicle, ^#^aPD1, or ^§^mpJX. n = 5–6 mice/group.

At 5 days, CD8^+^ cells assessed by immunohistochemistry were sparse after Vehicle and increased 2-fold after aPD1, 10-fold after mpJX, and 14-fold after mpJX+aPD1 (Figures 3E and 3F). 20 days after mpJX or mpJX+aPD1, CD8^+^ cells were still 6–7 times above baseline (Figures 3F and S3B). In contrast, few CD8^+^ cells or NKp46^+^ cells were found in normal pancreatic acini or liver of RT2;B6 mice treated with mpJX+aPD1. CD8^+^ cells were more than twice as numerous as NK cells 5 to 20 days after mpJX+aPD1 (Figure 4A). The persistence of CD8^+^ cells and, to lesser extent, NK cells in tumors over 20 days after mpJX+aPD1 (Figures 3D and 3F) accompanied an 85% suppression of tumor cell proliferation despite no vaccinia staining and a baseline level of apoptosis (Figures 1E and 3B).

The contributions of NK cells and CD8^+^ T cells to tumor cell killing after mpJX+aPD1 were further assessed by determining the effect of NK cell or CD8^+^ cell depletion in RT2;B6 mice.^16^ After NK cell depletion by an anti-NK1.1 antibody, apoptosis was not reduced 10 days after mpJX alone but was 39% less after mpJX+aPD1. By comparison, after CD8^+^ cell depletion by an anti-CD8 antibody, apoptosis was 53% less after mpJX and 77% less after mpJX+aPD1 (Figures 4B and 4C).

The distributions of NKp46^+^ cells and CD8^+^ cells differed markedly in tumors 5 days after mpJX+aPD1. NKp46^+^ cells were most numerous at focal sites of the vaccinia infection, but CD8^+^ cells were abundant both at those sites and in widespread regions of apoptosis (Figures 4D and 4E).

Recruitment of NK cells and CD8^+^ T cells was also compared to 7 other immune cell types by flow cytometric analyses of cells isolated from tumors in RT2;B6 mice 5 days after treatment onset. All cell types analyzed were sparse after Vehicle. CD8^+^ cells and B cells were significantly more numerous after aPD1 than after Vehicle, but all 9 cell types were more numerous after mpJX or mpJX+aPD1 (Figures 4F and S3C–S3F). After mpJX+aPD1, CD8^+^ cells were most abundant, followed by B cells, CD4^+^ cells, M1 macrophages, dendritic cells, M2 macrophages, NK cells, regulatory T cells, and NK T cells (Figure 4G; Table S1). CD8^+^ cells and NK cells were significantly more abundant after mpJX+aPD1 than after mpJX (Figure S3F and Table S1). Compared with the 2-fold greater number of CD8^+^ cells than NK cells found by immunohistochemical staining in tumor regions stained for the vaccinia antigen, CD8^+^ cells were more than 40 times as numerous as NK cells identified by flow cytometry among live/CD45^+^ cells isolated from whole tumors (Figure 4G; Table S1).

**Figure 5:**
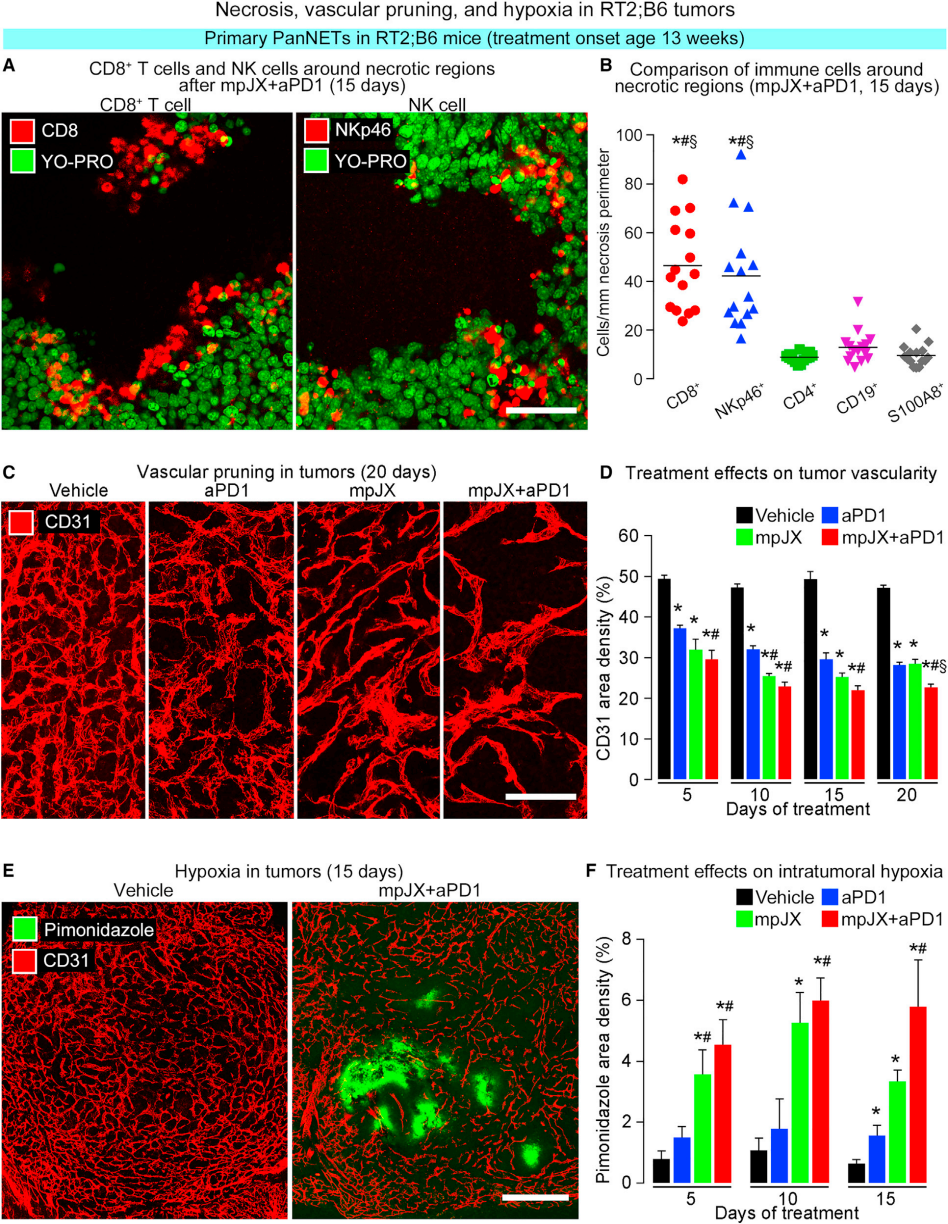
Necrosis, vascular pruning, and hypoxia in tumors of RT2;B6 mice (A) Confocal microscopic images showing numerous CD8^+^ cells (left) and NKp46^+^ cells (right) near the border of a necrotic region. Scale bar, 50 μm. (B) Measurements showing significantly more CD8^+^ cells and NKp46^+^ cells near necrotic regions than CD4^+^ cells, B cells (CD19^+^ cells), or neutrophils (S100A8^+^ cells) 15 days after mpJX+aPD1. ANOVA: p < 0.05 compared with ∗CD4^+^ cells, ^#^CD19^+^ cells, or ^§^S100A8^+^ cells. n = 15 mice/group. (C) Confocal microscopic images showing treatment-related changes in the vasculature (CD31, red) of RT2;B6 tumors at 20 days. Unlike the dense vasculature of the control tumor (Vehicle), vascularity is less after aPD1 or mpJX and even less after mpJX+aPD1. Scale bar, 100 μm. (D) Time-course of vascular pruning after treatment showing a significantly larger reduction after mpJX+aPD1. ANOVA: p < 0.05 compared with ∗Vehicle, ^#^aPD1, or ^§^mpJX. n = 5–12 mice/group. (E) Confocal microscopic images showing the absence of pimonidazole staining (hypoxia, green) in a Vehicle-treated tumor (left) compared with patches of strong staining in regions with sparse tumor vasculature (CD31, red) 15 days after mpJX+aPD1 (right). Scale bar, 400 μm. (F) Measurements comparing the amount of hypoxia 5 to 15 days after mpJX or mpJX+aPD1. Mean ± SEM. Student’s t test: p < 0.05 compared with ∗Vehicle or ^#^aPD1. n = 4–7 mice/group.

The types of immune cells abundant around necrotic regions were assessed by immunohistochemical staining 15 days after mpJX+aPD1 when necrosis was extensive. CD8^+^ cells and NK cells were similarly numerous near necrotic regions, but CD4^+^ cells, B cells, and neutrophils were sparse (Figures 5A and 5B). Together, the findings from immunohistochemical staining and flow cytometry show that CD8^+^ cells are the most abundant immune cells recruited to tumors after mpJX+aPD1, and the relative proportions of NK cells and other immune cells vary with the tumor region and timing of sampling.

### Vascular pruning, intratumoral hypoxia, and PD-LI expression

Consistent with previous evidence of the infection of tumors but not normal organs after i.v. administration of mpJX,^16^^,^^17^ we found patches of infection in tumors but not in normal pancreatic acini or in normal livers of RT2;B6 mice (Figure S4A).

Tumor vascularity, as assessed by CD31 staining, was reduced by 35% after mpJX and by 40% 5 days after mpJX+aPD1 (Figures S4B and S4C). Tumor vessels after mpJX or mpJX+aPD1 were reduced the most at 15 days (about 50% reduction) and at 20 days remained about the same after mpJX+aPD1 but increased significantly after mpJX (Figures 5C, 5D, and S4C). Surprisingly, tumor vascularity 20 days after aPD1 was 40% less than after Vehicle (aPD1 duration/vessel reduction linear regression R^2^ = 0.92, p *<* 0.05) (Figures 5C, 5D, and S4C).

The question of whether the reduction in tumor vascularity led to intratumoral hypoxia was addressed by injecting pimonidazole, which forms stable adducts in hypoxic cells that can be identified by immunohistochemistry.^24^ Although not present after Vehicle or aPD1, strong pimonidazole staining was found in poorly vascularized regions of tumors in RT2;B6 mice 5 to 15 days after mpJX and was even more extensive after mpJX+aPD1 (Figures 5E and 5F).

Because intratumoral hypoxia and oncolytic vaccinia viruses can increase the expression of PD-1 ligand PD-L1,^17^^,^^25^ we compared the amount and distribution of PD-L1 in tumors in RT2;B6 mice after treatment. PD-L1 staining was sparse after Vehicle or aPD1 but was much greater in some tumors 10 days after mpJX or mpJX+aPD1 (Figures S5A and S5B).

The presence of widespread vascular remodeling in tumors after mpJX+aPD1 raised the question of whether the vascular changes included the appearance of peritumoral high endothelial venules (HEVs) and lymphatics that could facilitate immune cell trafficking.^26^^,^^27^ MECA-79^+^ HEVs and LYVE1^+^ lymphatics were abundant around tumors of RT2;B6 mice after mpJX+aPD1, particularly when necrosis was extensive (Figures S5C and S5D), and were associated with CD8^+^ T cells (Figure S5E).

### Anti-metastatic action of mpJX+aPD1 in RT2;AB6F1 mice

#### Metastasis infection after i.v. injection of virus

**Figure 6:**
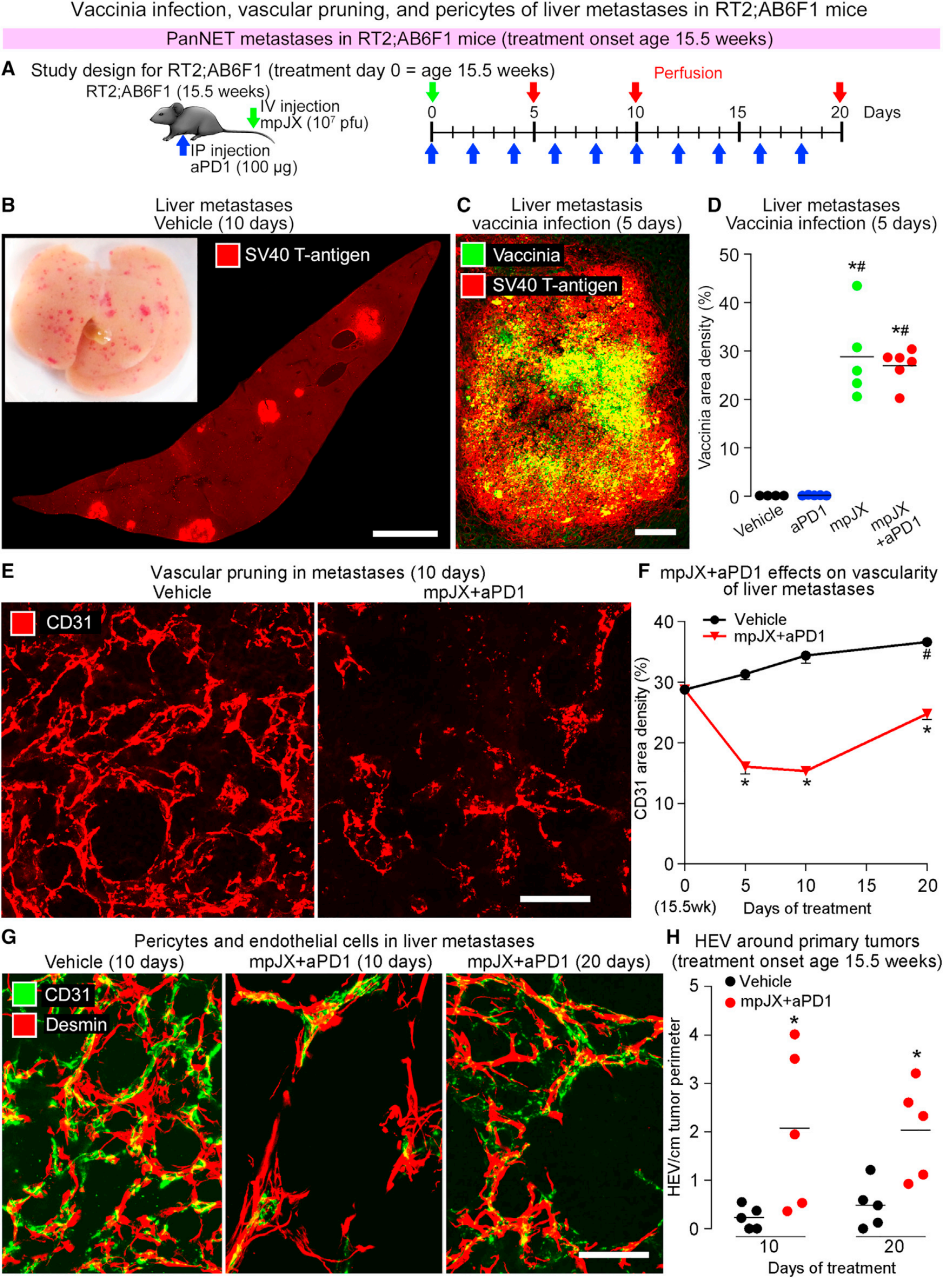
Treatment effects on liver metastases in RT2;AB6F1 mice (A) Study design showing that RT2;AB6F1 mice of both sexes at age 15.5 weeks received one i.v. dose of mpJX (10^7^ PFU) or Vehicle (PBS) on day 0 (green arrows) and i.p. injections of aPD1 (100 μg) or normal rat IgG2a every other day (blue arrows) for 5, 10, or 20 days (perfusion, red arrows). (B) Liver metastases visible as red spots on the surface (inset) and SV40 T-antigen staining (red) in histological section of RT2;AB6F1 mouse liver at age 17 weeks. Scale bar, 2.0 mm. (C) Confocal microscopic image of vaccinia infection (yellow-green) in RT2;AB6F1 liver metastasis (SV40 T-antigen, red) 5 days after mpJX+aPD1. Scale bar, 100 μm. (D) Dot plot of mean vaccinia area density in liver metastases in each RT2;AB6F1 mouse after mpJX (29%) or mpJX+aPD1 (27%). ANOVA: p < 0.05 compared with ∗Vehicle or ^#^aPD1. n = 4–6 mice/group. (E) Confocal microscopic images showing the dense vasculature (CD31, red) of a metastasis after Vehicle (left) and reduced vascularity 10 days after mpJX+aPD1 (right). Scale bar, 50 μm. (F) Time-course of reduction in metastasis vascularity over 20 days after mpJX+aPD1, while vascularity gradually increases after vehicle. Student’s t test: p < 0.05 compared with ∗Vehicle or ^#^Onset (day 0). n = 7–12 mice/group. (G) Confocal microscopic images of endothelial cells (CD31, green) and pericytes (desmin, red) in liver metastases after Vehicle and 10 and 20 days after mpJX+aPD1. Endothelial cells and pericytes were much less numerous 10 days after mpJX+aPD1 and somewhat more abundant at 20 days. However, pericytes were abnormally loosely associated with endothelial cells after Vehicle and at both times after mpJX+aPD1. No evidence of vascular normalization was found after mpJX+aPD1. Scale bar, 50 μm. (H) Dot plot showing greater abundance of HEVs around the perimeter of primary tumors in RT2;AB6F1 mice 10 and 20 days after mpJX+aPD1. Each dot is value for one mouse. Student’s t test: ∗p < 0.05 compared with Vehicle. n = 5 mice/group.

RT2;AB6F1 mice that spontaneously develop highly metastatic, poorly functional PanNETs^20^^,^^21^ were used to test the anti-metastatic activity of mpJX and mpJX+aPD1 at 5, 10, and 20 days (Figure 6A). Experiments designed with the same age and treatment duration revealed that primary PanNETs in RT2;B6 mice (Figures 2B–2F) and RT2;AB6F1 mice (Figures S6A–S6C) responded similarly to mpJX+aPD1.

The incidence of metastases visible as red or white spots on the liver surface of untreated RT2;AB6F1 mice (Figure 6B, inset) increased with age from 44% of mice at 15.5 weeks to 71% of mice at 17 weeks and 79% at 18.5 weeks (Table S2). Liver metastases identified microscopically as SV40/DAPI-stained cell clusters (Figure 6B), measuring up to 3.7 mm in diameter, were present in 56% of untreated RT2;AB6F1 mice at age 15.5 weeks, 93% at 17 weeks, and 93% at 18.5 weeks (Table S2). Suppression of this increase in metastasis was used as a metric of treatment efficacy.

Patches of vaccinia staining were present in all large metastases 5 days after mpJX or mpJX+aPD1 (Figure 6C) and occupied about 27% of the sectional area (Figure 6D), indicative of direct infection of metastases after i.v. injection of the virus and of no suppression of infection by aPD1. This index of infection of metastases exceeded corresponding values of 13% in primary tumors in RT2;AB6F1 mice 5 days after mpJX+aPD1 (Figures S6D and S6E) and 6% in RT2;B6 mice (Figure 3B).

#### Vascular abnormalities in metastases

Blood vessels in liver metastases were abundant and as abnormal as in the primary tumors of RT2;AB6F1 mice. The vascular density in metastases increased significantly (27%) over the 20-day study (Figures 6E and 6F). After mpJX+aPD1, metastasis vascularity was reduced by 49% at 5 days, 55% at 10 days, and 32% at 20 days, compared with the corresponding values for Vehicle (Figures 6E and 6F). Loss of vascular endothelial cells in metastases was accompanied by similar reductions in pericytes (Figure 6G). Pericytes had an abnormally loose association with blood vessels regardless of treatment (Figure 6G), indicating that the pericyte abnormality in metastases was not reversed by mpJX+aPD1, consistent with evidence that mpJX treatment does not result in vascular normalization in PanNET.^16^

Although HEVs were more numerous in primary tumors of RT2;AB6F1 mice treated with mpJX+aPD1 (Figure 6H), as in RT2;B6 mice (Figures S5C–S5E), no peritumoral HEVs or lymphatics were identified around liver metastases in RT2;AB6F1 mice regardless of the treatment over 10 or 20 days.

#### NK cell and CD8 T cell influx in metastases

**Figure 7:**
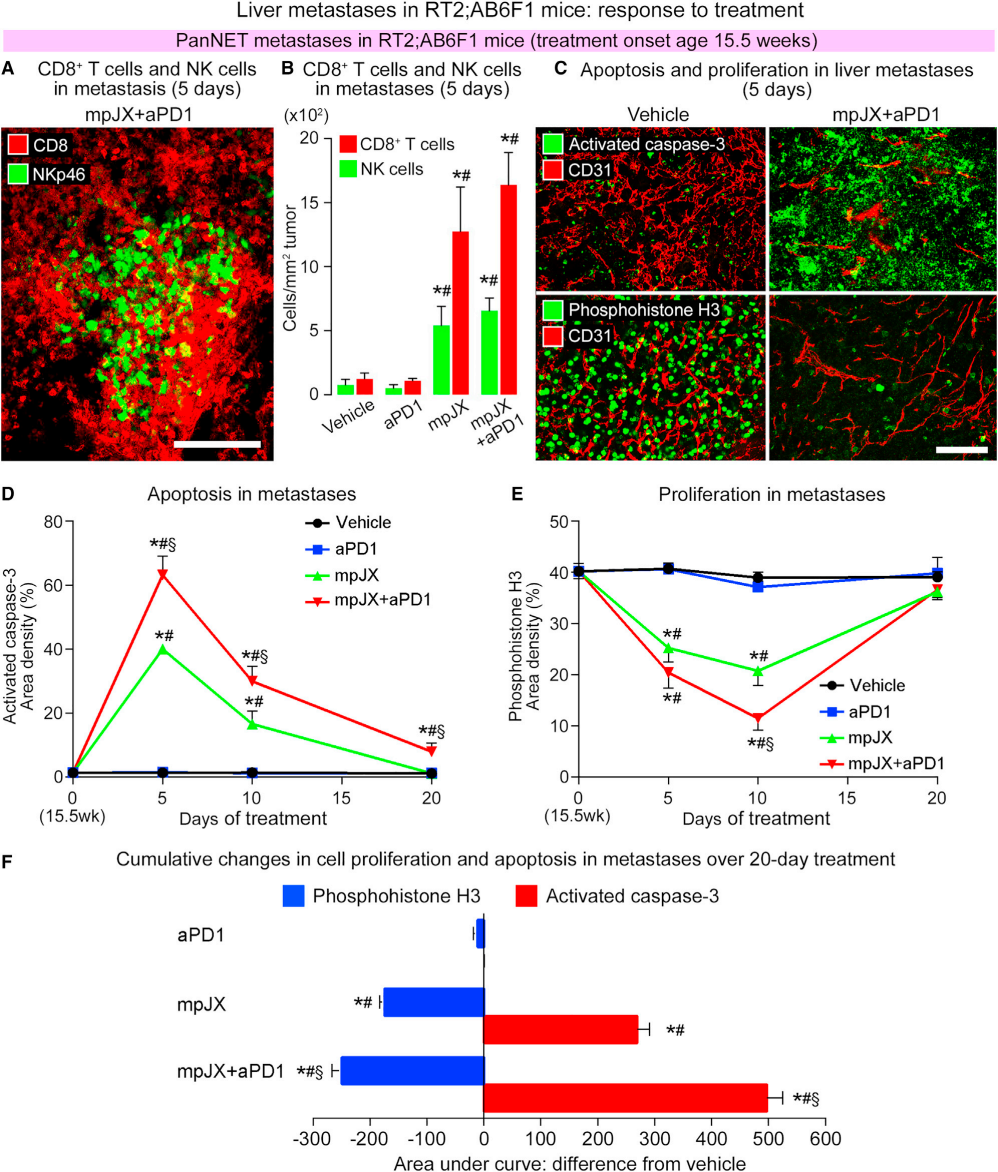
Influx of CD8^+^ T cells and NK cells, increased apoptosis, and reduced proliferation in liver metastases in RT2;AB6F1 mice after treatment (A) Confocal microscopic image of scattered NK cells (NKp46, green) and more abundant CD8^+^ T cells (CD8, red) in metastasis 5 days after mpJX+aPD1. Scale bar, 100 μm. (B) Measurements showing more CD8^+^ T cells than NK cells in metastases after mpJX or mpJX+aPD1. ANOVA: p < 0.05 compared with ∗Vehicle or ^#^aPD1. n = 4–5 mice/group. (C) Confocal microscopic images of metastases showing more apoptosis (upper row, activated caspase-3, green) and less proliferation (lower row, phosphohistone H3, green) 5 days after mpJX+aPD1 (right) than after Vehicle (left). Blood vessels (CD31, red). Scale bar, 100 μm. (D) Line plots showing 5-day peak of activated caspase-3 in metastases after mpJX (40% area density) and even more after mpJX+aPD1 (63% area density). (E) Line plots showing reduction of phosphohistone H3 that was greatest 10 days after mpJX (48% reduction) or mpJX+aPD1 (71% reduction). ANOVA: p < 0.05 compared with ∗Vehicle, ^#^aPD1, or ^§^mpJX. n = 4–8 mice/group. (F) Calculated areas under the curves in (D) and (E) showing the cumulative decrease in metastasis proliferation and the increase in apoptosis over 20 days normalized to the value for Vehicle (area = 0). The decrease in proliferation and increase in apoptosis in metastases after mpJX+aPD1 were significantly greater than after mpJX. (D–F) ANOVA: p < 0.05 compared with ∗Vehicle, ^#^aPD1, or ^§^mpJX. n = 5 mice/group.

Immunohistochemical staining revealed that NKp46^+^ cells and CD8^+^ cells were sparse or absent in metastases in RT2;AB6F1 mice after Vehicle or aPD1 but were abundant after mpJX or mpJX+aPD1 (Figures 7A and 7B). As in primary tumors, CD8^+^ cells were more than twice as abundant as NKp46^+^ cells after mpJX or mpJX+aPD1 (Figure 7B).

#### Tumor cell apoptosis and proliferation in metastases

Comparison of treatment effects on tumor cell killing in metastases in RT2;AB6F1 mice revealed that apoptosis was sparse after Vehicle (1.4% area density) and changed little after aPD1 (1.5% area density; Figures 7C and 7D) but was extensive 5 days after mpJX (40% area density) and even more widespread after mpJX+aPD1 (63% area density) (Figure 7D). Although apoptosis was less at 10 and 20 days (Figure 7D), the amount was significantly greater after mpJX+aPD1 than after mpJX at both time points (Figure 7D). In contrast, proliferating cells in metastases were abundant after Vehicle (40% area density; Figures 7C and 7E) and changed little after aPD1 but were less numerous at 10 days after mpJX (48% reduction) and were even fewer at 10 days after mpJX+aPD1 (71% reduction) and then returned to the baseline level at 20 days (Figure 7E).

Calculation of the cumulative changes in apoptosis and proliferation in metastases over the 20-day study, reflected by the area under the curves compared with Vehicle, showed that tumor cell killing increased 14-fold after mpJX and 25-fold after mpJX+aPD1, whereas proliferation decreased 29% after mpJX and 42% after mpJX+aPD1 (Figure 7F). Both changes were significantly larger after mpJX+aPD1 than after mpJX (Figure 7F).

#### Reduction in metastasis number, size, and burden

**Figure 8:**
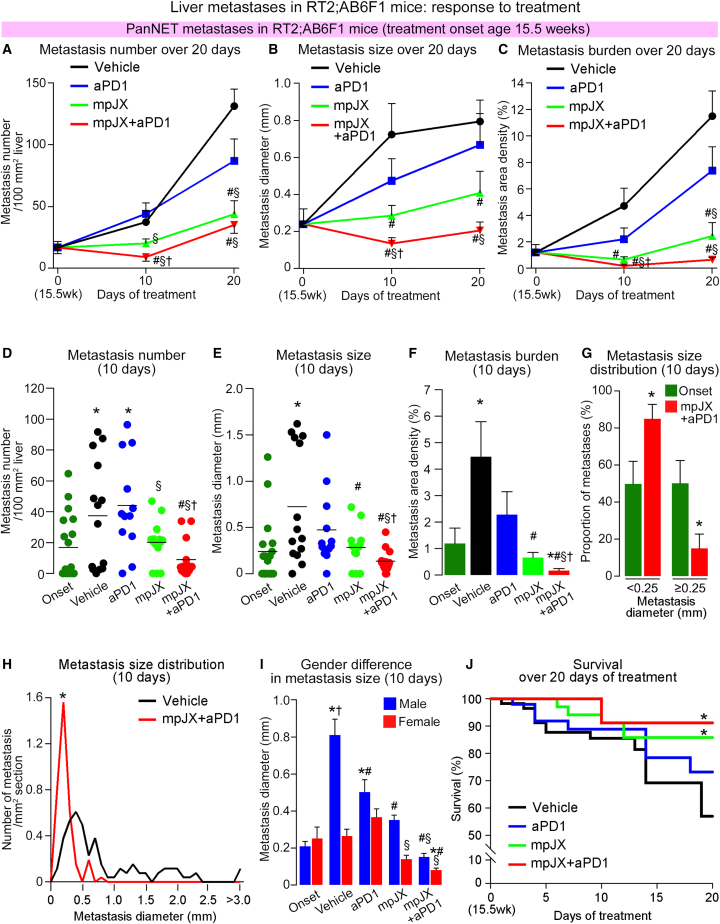
Reduction in number and size of liver metastases in RT2;AB6F1 mice after treatment (A–C) Line plots showing significantly larger changes in metastasis number (A), size (B), and burden (C) after mpJX+aPD1 over 10 or 20 days. (D–F) Plots showing fewer (D) and smaller (E) metastases and less metastatic burden (F) 10 days after mpJX+aPD1 than after other treatments. Dots show the mean for each mouse. Metastatic burden calculated from metastasis number and size. (A–F) Student’s t test: p < 0.05 compared with ∗Onset, ^#^Vehicle, ^§^aPD1, or ^†^mpJX. n = 12–18 mice with or without metastases/group (both sexes). (G) Diameter frequency distribution documenting metastasis regression, where metastasis size was smaller 10 days after mpJX+aPD1 than at onset. Student’s t test: ∗p < 0.05 compared to onset. n = 10 mice with metastases in onset group and 12 mice with metastases in mpJX+aPD1 group. (H) Line plots of size distributions showing uniformly smaller metastases 10 days after mpJX+aPD1 than after Vehicle. Kolmogorov-Smirnov test: p < 0.001 compared with ∗Vehicle. n = 109 metastases in 13 mice after Vehicle and 88 metastases in 12 mice after mpJX+aPD1. (I) Sex differences in metastasis diameter showing larger metastases in Vehicle-treated males (10 days). ANOVA: p < 0.05 compared with ∗Onset, ^#^Vehicle, ^§^aPD1, or ^†^Females. All metastases in 5–12 mice with or without metastases of each sex per group. (J) Kaplan-Meier plots showing greater survival 20 days after mpJX+aPD1 (91%) than after mpJX (86%), aPD1 (73%), or Vehicle (57%). Log-rank test: ∗p < 0.05 compared with Vehicle. n = 49–57 mice/group.

Liver metastasis progressed rapidly in RT2;AB6F1 mice during the 20-day study (Table S2). Compared with the experiment onset at age 15.5 weeks, metastases in Vehicle-treated controls increased in number about 2-fold over 10 days and 7-fold over 20 days and increased in diameter about 3-fold at 10 days and 4-fold at 20 days (Figures 8A and 8B; Table S3).

Treatment with mpJX or mpJX+aPD1 had large and sustained effects on metastasis number, size, and burden, but aPD1 alone had relatively little effect (Figures 8A–8C; Table S3). Compared with Vehicle, the largest reductions in all three readouts were found after mpJX+aPD1, where metastases were 76% less numerous and 81% smaller at 10 days and 71% less numerous and 74% smaller at 20 days (Figures 8A–8F; Table S3).

Overall metastatic burden (mm^2^ metastasis area/mm^2^ liver section area), which reflected both the number and size of metastases, was 96% less after mpJX+aPD1 than after Vehicle at 10 days and 94% less at 20 days (Figures 8A–8C; Table S3). To test whether the number of mice that had no metastases at the end of treatment influenced these values, the metastatic burden was calculated separately for mice with metastases and mice without metastases. The results were similar. Metastatic burden in mice with metastases present after treatment with mpJX+aPD1 was 96% less at 10 days and 93% less at 20 days than after Vehicle (Figure S6F; Table S4).

The question of whether treatment not only slowed metastasis growth but also caused regression was addressed by comparing metastasis size after mpJX+aPD1 to corresponding values at the onset of treatment. This comparison revealed that metastases were 65% smaller 10 days after mpJX+aPD1 than in the onset group, indicative of regression (Figure 8G). Reduction in metastasis size was evident across the entire size range (Figures 8H and S6G). A similar comparison 20 days after mpJX+aPD1 revealed that metastases had enlarged and were no longer significantly smaller than the onset group but were still on average only one-quarter the diameter of Vehicle-treated controls (Figure S6G; Tables S3 and S4).

Compared with mice receiving mpJX alone, metastases were 55% less numerous and 56% smaller and metastasis burden was 73% less 10 days after mpJX+aPD1 (Figures 8D–8F; Table S3), consistent with larger effects of mpJX+aPD1 on CD8^+^ cell influx, tumor cell apoptosis, and suppression of proliferation (Figure 7F; Table S1). Differences at 20 days were in the same direction but smaller (Figures 8A–8C; Table S3).

Although the foregoing data were from mouse cohorts balanced for sex, we also evaluated metastasis number, size, and burden separately in both sexes because male RT2;AB6F1 mice are reported to have more aggressive liver metastasis.^28^ Liver metastases were indeed 3 times larger on average in males at age 17 weeks, but both sexes responded similarly to mpJX and mpJX+aPD1 (Figure 8I). Significant sex differences were not found in treatment effects on metastasis number, size, or burden (Figures S7A–S7C), although detection of small differences would require larger cohorts.

Survival of RT2;AB6F1 mice over the 20-day study was examined in cohorts of 49–57 mice in each of the four treatment groups. Survival at 20 days was significantly greater in mice that received mpJX+aPD1 (91%) or mpJX alone (86%) than Vehicle (57%) (Figure 8J).

Together, these results provide evidence that liver metastases were reduced in number, size, and overall burden by one i.v. dose of mpJX together with repeated doses of aPD1 every other day for 10 or 20 days. Metastatic burden at 10 days after mpJX+aPD1 was even less than at the onset, reflecting regression of existing liver metastases.

## Discussion

With the goal of determining the influence of i.v. administration of the vaccinia virus mpJX on the antitumor activity of PD-1 blockade on PanNETs that are immunologically cold and either functional or metastatic, we used two contrasting genetic mouse models: one that develops non-metastatic, insulin-secreting PanNETs, and another that develops poorly functional primary tumors and abundant liver metastases. Experiments revealed that one i.v. dose of mpJX administered with repeated doses of the anti-PD-1 antibody infected both primary tumors and metastases and had synergistic effects on the influx of NK cells, CD8^+^ T cells, and other immune cells and on tumor cell killing and suppression of tumor cell proliferation. Insulin secretion and hypoglycemia were reduced and survival prolonged in mice with functional PanNETs, and metastatic tumor burden was decreased to less than the onset of treatment in mice with metastatic PanNETs.

### Approach for comparing antitumor activity

Experiments were designed to determine the relative contributions of mpJX and aPD1 to antitumor activity on spontaneous functional or metastatic PanNETs. The studies built on evidence that mpJX administered i.v. is efficacious in RT2;B6 mice with functional PanNETs,^16^ raising the potential for direct anti-metastatic effects of the virus in RT2;AB6F1 mice independent of abscopal changes after i.t. injection.^10^^,^^12^^,^^29^ Studies designed to evaluate separately the responses to the virus and to the PD-1 blockade revealed the evolution of antitumor activity of each individually and together in the context of the immune response, vascular changes, tumor functionality, and metastasis. By design, one dose of the virus was used as a reference for future studies of repeated dosing on PanNETs and other tumor types in mice^16^ and patients.^30^

Although mpJX expresses hGM-CSF,^6^ which has limited activity in mice,^31^^,^^32^ mpJX produces amounts of tumor cell killing at 5 days equivalent to engineered variants that express mGM-CSF or no GM-CSF.^16^ Expression of mGM-CSF by another WR strain vaccinia virus similar to mpJX increased tumor cell killing,^17^ but the replacement of hGM-CSF with mGM-CSF in Wyeth strain JX-594 did not increase antitumor efficacy or survival despite a greater inflammatory response in a rodent glioblastoma.^33^ Use of the anti-GM-CSF antibody to test the contribution of viral mGM-CSF^10^ introduces the complication of blocking endogenous non-viral mGM-CSF. Notwithstanding the potent antitumor activity of mpJX, the magnitude of the contribution of viral GM-CSF expression to efficacy deserves further study.

A central feature of the experimental design was the comparison of sequential cellular changes in functional and metastatic PanNETs. RT2;B6 mice develop functional PanNETs characterized by tumor insulin secretion, hypoglycemia, and premature mortality but rare metastases.^16^^,^^18^^,^^19^^,^^22^^,^^23^ By comparison, the F1 offspring of RT2;B6 males bred with wild-type A/J females (RT2;AB6F1 mice) develop poorly functional, highly metastatic PanNETs.^19^, ^20^, ^21^^,^^28^ Experiments revealed that primary PanNETs responded similarly to mpJX+aPD1 in RT2;B6 mice and RT2;AB6F1 mice. Treatment of RT2;B6 mice resulted in decreased viable tumor cell mass, lowered insulin secretion, and increased blood glucose. Treatment of RT2;AB6F1 mice also led to mpJX infection of metastases and reduced metastatic burden. Because metastasis is more aggressive in males,^28^ RT2;AB6F1 mice enabled delineation of sex differences in treatment efficacy.

The presence of necrosis confounded the interpretation of the overall size of larger tumors. The finding of little or no size reduction in the largest tumors with necrotic regions—but clear reduction in smaller tumors—could reflect pseudo-progression, where overall tumor size can stay the same or increase when necrosis, immune cells, and edema make significant contributions to tumor mass.^34^^,^^35^

### Factors contributing to greater antitumor action of mpJX+aPD1

Six factors considered as potential contributors to the greater efficacy of mpJX+aPD1 on functional PanNETs were: (1) the oncolytic action of the virus itself; (2) apoptosis and suppression of tumor cell proliferation; (3) the influx of NK cells, CD8^+^ T cells, and other immune cells; (4) vascular changes including tumor vessel pruning, intratumoral hypoxia, necrosis, and development of HEVs and lymphatics; (5) upregulation of PD-L1; and (6) reduced tumor insulin secretion.

#### Direct oncolytic activity

Tumor cell killing by viral lysis is a well-documented feature of vaccinia virus infection.^7^^,^^10^^,^^36^ However, the limited sites of tumor infection (6% area) compared with killing (>60%) and similar amount of infection despite greater killing after mpJX+aPD1 than after mpJX alone argue against oncolytic activity explaining the amplified antitumor activity of mpJX+aPD1.

#### Widespread apoptosis and suppression of proliferation

A key discrepancy in weighing the contribution of direct viral lysis is evident from the amount of apoptosis in tumors that far exceeds the regions of infection after i.v. administration of the virus.^16^^,^^17^^,^^37^ Apoptosis occupied 5 times the tumor area of the vaccinia infection 5 days after mpJX and 10 times the area after mpJX+aPD1. Doubling of apoptosis by combining mpJX with aPD1 implicates mechanisms other than viral lysis.

Viral oncolytic activity also does not explain the widespread and progressive suppression of tumor cell proliferation. Unlike focal regions of the vaccinia infection in tumors, dividing cells were reduced by 59% throughout tumors 20 days after mpJX and reduced by 85% after mpJX+aPD1. Vaccinia viruses can reduce tumor cell proliferation *in vitro*^38^ and *in vivo*,^39^ but unlike direct actions of chemotherapeutic agents on the cell cycle,^40^^,^^41^ the anti-mitotic action of vaccinia viruses is likely to be indirect. Vaccinia viruses increase interferon-gamma (IFN-γ) gene expression in tumors.^17^ IFN-γ suppresses tumor cell proliferation^42^^,^^43^ and contributes to tumor growth suppression after vaccinia viruses, as shown by studies of IFN-γ-knockout mice^29^ or IFN-γ-blocking antibody.^8^^,^^12^^,^^44^

#### CD8^+^ T cells, NK cells, and other immune cells

Innate and adaptive immune responses contribute to tumor cell apoptosis and arrest of proliferation after vaccinia viruses.^8^^,^^29^^,^^44^ Increases in 8 types of immune cells were found in RT2;B6 tumors 5 days after mpJX+aPD1, with CD8^+^ cells, B cells, and CD4^+^ cells beingmost numerous.

The magnitude of the CD8^+^ cell increase after mpJX+aPD1, compared with mpJX alone, reflected synergistic effects of mpJX and aPD1. The greater action of mpJX+aPD1 fits with aPD1 promotion of immune cell recruitment and expansion of CD8^+^ T cell antitumor activity^11^ and with continued CD8^+^ T cell influx during PD-1 blockade over 20 days after one dose of mpJX. Unlike NK cells, CD8^+^ cells were as widely distributed as apoptosis. Tumor cell killing after vaccinia virus infection is accompanied by large increases in expression of CD8^+^ T cell cytotoxicity genes granzyme A, granzyme B, Fas ligand, and perforin-1.^17^

CD8^+^ T cell contribution to widespread apoptosis after mpJX+aPD1 is further supported by the reduction of the synergistic action by CD8^+^ cell depletion. These findings fit with other evidence for reduced antitumor activity of vaccinia viruses after CD8^+^ cell depletion.^9^^,^^10^^,^^12^^,^^16^^,^^29^^,^^44^ By comparison, NK cell depletion has mixed effects on tumor growth suppression, and CD4^+^ cell depletion has little or no effect.^8^^,^^9^^,^^12^^,^^29^^,^^44^ However, as apoptosis was not abolished by CD8^+^ cell depletion, additional mechanisms are likely to contribute to the greater antitumor activity of mpJX+aPD1.

Although NK cells were more abundant in tumors 5 days after mpJX+aPD1 than after mpJX or aPD1 alone and remained higher over 20 days, NK cells accumulated at sites of the vaccinia infection after 5 days but were sparse in other regions of apoptosis. At 15 days, NK cells and CD8^+^ cells were the most abundant immune cells detected around necrotic regions of tumors. NK cell depletion reduces oncolytic virus-mediated tumor growth suppression in some models,^9^^,^^29^ and PD-1 inhibition prolongs the NK cell response,^45^ yet the role of NK cells is complicated by actions against viruses and multiple host cell types.^46^

#### Vascular pruning, HEVs, and lymphatics

Vaccinia viruses have striking effects on tumor vasculature in mice and humans.^10^^,^^16^^,^^17^^,^^36^^,^^37^^,^^39^^,^^47^ After i.v. injection, the virus infects and disrupts tumor endothelial cells before infecting tumor cells.^16^^,^^37^ Vascular pruning was greater after mpJX+aPD1 than after mpJX alone, was sustained for at least 20 days, and was not accompanied by vascular normalization. Reduced vascularity was accompanied by intratumoral hypoxia and necrosis, as in previous reports.^47^

Vascular pruning after mpJX+aPD1 was accompanied by the development of peritumoral HEVs and lymphatic vessels over 10 days. HEV formation facilitates CD8^+^ T cell influx and improves antitumor immunity after PD-1 blockade.^26^^,^^27^ As vascular remodeling can promote immune cell infiltration,^48^^,^^49^ mpJX+aPD1 effects on the tumor vasculature deserve further study.

As reported in previous studies of mpJX and other oncolytic vaccinia viruses,^16^^,^^17^ i.v. injection of mpJX did not result in infection of endothelial cells of normal blood vessels, and immune cells were not recruited to normal regions of the pancreas or liver.

#### PD-L1 upregulation

Building on evidence that PD-L1 expression is increased by HIF-1α,^25^^,^^50^ we found that vascular pruning and intratumoral hypoxia after mpJX+aPD1 were accompanied by increased PD-L1 immunoreactivity. Upregulation of PD-L1 after mpJX+aPD1 also implicates IFN-γ from CD8^+^ T cells and other immune cells that promote PD-L1 expression after viral infection.^51^^,^^52^ As potential translational significance, increased PD-L1 is positively correlated with successful antitumor immunity and patient response to checkpoint inhibition.^53^^,^^54^

#### Tumor insulin secretion and survival

Stabilization of blood insulin and blood glucose by mpJX+aPD1 contributed to the prolongation of survival of mice with functional PanNETs. Decreased insulin secretion and less hypoglycemia are likely consequences of the reduction in viable tumors. As hypoglycemia is a risk factor in these mice, blood insulin and glucose served as prognostic biomarkers. Treatment with mpJX+aPD1 increased survival to age 16 weeks from 43% to 73%, a 70% improvement.

### Regression of metastases after mpJX+aPD1

One of the most striking findings in our study was the regression of liver metastases in RT2;AB6F1 mice after mpJX+aPD1. Although mpJX administered alone reduced the number and size of metastases, mpJX+aPD1 had significantly greater activity and reduced overall metastatic burden by 96% compared with Vehicle-treated controls and by 85% compared with onset controls at 10 days. Incidence of metastasis was also reduced, albeit not to zero, but at 10 days, the size and number of metastases were reduced to less than at the beginning of treatment. Metastatic burden 20 days after mpJX+aPD1 was similar to the onset and was 93% lower and survival was 60% greater than in mice treated with Vehicle.

Anti-metastatic effects on implanted or injected human or mouse tumor cells have been reported after i.v., i.t., or intraperitoneal (i.p.) injection of vaccinia viruses.^10^^,^^12^^,^^29^^,^^39^ Similarly, fewer lung metastases were found in a prevention study after i.t. injection of a vaccinia virus plus aPD1 and anti-CTLA-4 into MMTV-PyMT mice with mammary carcinomas.^10^ Although i.t. administration of a vaccinia virus combined with aPD1 has abscopal growth-slowing activity on a second implanted tumor,^10^^,^^12^ still unknown until the current study was whether a vaccinia virus plus aPD1 promoted regression of existing liver metastases that develop from spontaneous tumors and recapitulate aggressive human PanNETs.^5^^,^^55^^,^^56^

To address these issues, we evaluated anti-metastatic activity in the context of tumor targeting and changes in tumor cells and immune cells in PanNET metastases in RT2;AB6F1 mice. Importantly, metastases, like primary tumors, were infected after i.v. injection of mpJX. Although the level of infection was similar to mpJX alone, mpJX+aPD1 promoted a greater influx of NK cells and CD8^+^ T cells, tumor cell killing, suppression of proliferation, and regression of metastases. Metastatic burden was 74% less than after mpJX alone when assessed by a histopathological metric that correlates closely with 3-dimensional bioluminescence imaging.^57^ Also important was the restriction of patches of vaccinia infection, influx of NK cells and CD8^+^ T cells, and widespread apoptosis to metastases, with few or none in normal regions of the liver and pancreas. Furthermore, mpJX+aPD1 reduced metastasis with similar efficacy in both sexes despite the presence of larger and more aggressive tumors in male RT2;AB6F1 mice.^28^ Evidence of mpJX+aPD1 efficacy at reversing existing metastases is timely because of ongoing clinical trials of vaccinia viruses with checkpoint inhibitors in patients with metastatic disease (ClinicalTrials.gov: NCT03294083 and NCT03206073).

### Conclusions

Administration of one i.v. dose of vaccinia virus mpJX with repeated dosing of aPD1 had synergistic effects on the influx of NK cells, CD8^+^ T cells, and other immune cells and on tumor cell apoptosis and suppression of tumor cell proliferation in two contrasting genetic mouse models of PanNETs. Synergistic actions of mpJX and aPD1 led to stabilization of blood insulin and glucose and reduced mortality of mice with functional PanNETs. The combination of mpJX and aPD1 also decreased metastatic burden to less than the beginning of treatment in mice with metastatic PanNETs. Together, the findings support the rationale for combining oncolytic vaccinia viruses with PD-1 blockade in the treatment of functional or aggressive PanNETs.

## Experimental procedures

### Oncolytic virus

Vaccinia virus mpJX-594 (mpJX) was engineered at the Ottawa Hospital Research Institute from the WR strain using the same plasmid design as Wyeth strain JX-594.^16^ WR is a Wyeth strain vaccinia virus isolated through serial passage in mice to select for replication in mouse cells.^6^ A cassette containing enhanced green fluorescent protein (EGFP) and hGM-CSF transgenes under the synthetic early/late promoter (pSE/L) was inserted into the vaccinia thymidine kinase gene locus to inactivate the gene function.^16^

### PanNET models and treatments

PanNET-bearing RT2;B6 mice^18^^,^^19^^,^^21^ were assigned to four groups at age 13 weeks. The Vehicle group received one i.v. dose of phosphate-buffered saline (PBS) and sequential doses of normal rat IgG2a (BioXCell BE0089, 100 μg in 200 μL PBS, West Lebanon, NH, USA) by i.p. injection. The aPD1 group received one i.v. dose of PBS and sequential i.p. doses of aPD1 (BioXCell RMP1-14, 100 μg in 200 μL PBS). The mpJX group received one i.v. dose of virus (10^7^ PFU in 100 μL PBS) and sequential i.p. doses of IgG2a. The mpJX+aPD1 group received both mpJX and aPD1. Mice received the virus or PBS by tail-vein injection on the morning of day 0 and aPD1 or control IgG2a by i.p. injection the same afternoon and every other day over the 5-, 10-, 15-, or 20-day study duration. Onset controls were studied at age 13 weeks. CD8^+^ T cells and NK cells were depleted in some mice by injections of an anti-CD8 antibody or an anti-NK1.1 antibody before and during the treatment (details in supplemental information).

RT2;AB6F1 hybrid mice, which have PanNETs that develop abundant liver metastases and secrete less insulin, were bred from RT2;B6 males and wild-type A/J females.^20^^,^^28^ F1 offspring were treated for 5, 10, or 20 days beginning at age 15.5 weeks, except in the age-matched comparison of treatment-induced necrosis in primary tumors of RT2;B6 mice and RT2;AB6F1 mice at age 13 weeks. Onset controls were studied at age 15.5 weeks (details in supplemental information). All mice were housed under barrier conditions in the animal care facility at the University of California, San Francisco (UCSF). All experimental procedures were approved by the Institutional Animal Care and Use Committee.

### Blood glucose and insulin

Mice with free access to water were fasted for 3 h in the morning of day 0 and again at the end of treatment. Glucose in freshly drawn blood was measured by ACCU-CHEK Performa (Roche). Serum insulin was measured in a second blood sample by ELISA (details in supplemental information).

### Tissue preparation and immunohistochemistry

After the final blood collection for glucose and insulin measurements, some mice received pimonidazole (1.5 mg in 100 μL PBS) by i.p. injection 1 h before vascular perfusion to assess intratumoral hypoxia. After mice were anesthetized (ketamine 87 mg/kg and xylazine 13 mg/kg by i.p. injection), tissues were preserved by vascular perfusion of 1% paraformaldehyde (PFA) in PBS. The pancreas and liver were removed, embedded in optimal cutting temperature (OCT) compound, frozen, and prepared for immunohistochemical staining.^16^^,^^58^

Cryostat sections 80 μm in thickness were stained with antibody combinations for immunohistochemical assessment of viral antigen (vaccinia); tumor cells (SV40 T-antigen, SV40); apoptosis (activated caspase-3); proliferation (phosphohistone H3); CD8^+^ T cells (CD8 antigen); CD4^+^ T cells (CD4); B cells (CD19); NK cells (NKp46^59^); PD-L1; blood vessels in primary tumors and metastases (CD31); normal liver sinusoids (VEGFR2); pericytes (desmin); HEVs (MECA-79); lymphatic vessels (LYVE-1); and intratumoral hypoxia (pimonidazole). Primary antibodies were localized with species-specific secondary antibodies. Cell nuclei were stained with TO-PRO-1, YO-PRO-1, or DAPI. Specimens were examined with a Zeiss Axiophot fluorescence microscope with an Olympus DP73 camera and with a Zeiss LSM 510 laser scanning confocal microscope^58^ (details in supplemental information).

### Morphometric measurements

The size of primary tumors in RT2;B6 and RT2;AB6F1 mice was measured 15 days after the beginning of treatment at age 13 weeks in images of the 10 largest tumors in a section of each pancreas stained for SV40, CD31, and DAPI by tracing the tumor perimeter in ImageJ (version 1.52s, https://imagej.nih.gov/ij/). The number of pixels was converted to square millimeters, and tumor diameter (mm) was calculated from the area assuming circularity. Regions of intratumoral necrosis, identified by the absence of DAPI staining (Figure S1D), were measured in images of the 5 largest tumors and expressed as a proportion of total tumor area. Viable tumor area was calculated from total tumor area minus necrosis.

Fractional area (area density, %) of immunohistochemical staining for vaccinia, activated caspase-3, phosphohistone H3, CD31, pimonidazole, and PD-L1 was measured in fluorescence microscopic images of 80 μm sections of the 5 largest tumors (diameter >1.5 mm) or metastases in RT2;B6 or RT2;AB6F1 mice. Cytotoxic T cells, identified as CD8^+^ cells, and NK cells, identified as NKp46^+^ cells, were counted in confocal microscopic images of the 5 largest tumors or metastases and expressed as numerical densities (cells/mm^2^). CD8^+^ T cells, NK cells, CD4^+^ cells, B cells (CD19^+^ cells), and neutrophils (S100A8^+^ cells) near the border of necrotic regions in tumors were counted in confocal microscopic images of the 5 largest tumors and expressed as numerical densities (cells/millimeter perimeter of necrotic region) (details in supplemental information).

Metastases were analyzed in sections of RT2;AB6F1 mouse liver in cryostat blocks containing 7 pieces: 3 pieces of left lobe, 2 pieces of left median lobe, and 2 pieces of right median lobe (Figure S8A). Metastases were identified as SV40^+^ tumor-cell clusters measuring 50 μm or larger in diameter. When SV40 staining was weak, metastases were identified as collections of densely packed nuclei stained by DAPI or TO-PRO-1, which gave identical values in validation studies (Figures S8B–S8E). The number of metastases was expressed per 100 mm^2^ of liver sections. Sectional areas of the 10 largest metastases were measured and expressed as diameters, as for primary tumors. Mice without liver metastases were assigned zero values for number and size. Values for males and females were analyzed separately because of the sexual dimorphism of metastasis in RT2;AB6F1 mice.^28^ Metastatic tumor burden was calculated as the total area of metastases per area of liver sections (mm^2^/mm^2^) and expressed as an area density (%). Others have designated this value the “hepatic replacement area,” which correlates closely with the 3-dimensional metastatic tumor burden assessed by luciferase bioluminescence imaging.^57^ HEVs around the 5 largest primary tumors in RT2;AB6F1 mice were counted by confocal microscopic examination and expressed as numerical densities (HEVs/centimeter tumor circumference) (details in supplemental information).

### Flow cytometry analysis

Immune cell influx into tumors in RT2;B6 mice after treatment over 5 days was assessed by flow cytometry. Tumors were removed from anesthetized mice after perfusion with PBS, weighed, and digested. Dissociated cells were stained for flow cytometric analysis of CD8^+^ cells, NK cells, CD4^+^ cells, B cells, regulatory T cells, NK T cells, dendritic cells, and M1 and M2 macrophages (BD LSR Fortessa and FlowJo software) (details in supplemental information).

### Statistical analysis

Mice of both sexes matched for age were randomly assigned to groups. Group size was determined by power analysis of data from pilot studies to achieve statistical power of 0.8 and a p value of 0.05. Sex was tested as a biological variable with special attention given to gender differences in liver metastasis in RT2;AB6F1 mice.^28^ Values are expressed as mean ± SEM for each group, where the number of mice per group is shown in the figure legends. Differences were assessed by one-way ANOVA followed by Tukey test for multiple comparisons or Student’s t test (Prism 8, GraphPad). Mann-Whitney U or Kolmogorov-Smirnov two-sample tests were used where data were not normally distributed. Differences in Kaplan-Meier plots of survival distributions were assessed by the log rank test.

## Supplemental information

Document S1. Supplemental methods, Figures S1–S8, and Tables S1–S4

Document S2. Article plus supplemental information

## Footnotes

Supplemental information can be found online at https://doi.org/10.1016/j.omto.2021.12.016.
