# Supplementary material for: Oncolytic vaccinia virus injected intravenously sensitizes pancreatic neuroendocrine tumors and metastases to immune checkpoint blockade
Source: Mol Ther Oncolytics. 2021 Dec 21;24:299–318. doi: 10.1016/j.omto.2021.12.016 (PMC8783073; doi:10.1016/j.omto.2021.12.016)
Supplement: Document S1. Supplemental methods, Figures S1–S8, and Tables S1–S4 [file mmc1.pdf]

OMTO, Volume *24*

Supplemental information

Oncolytic vaccinia virus injected intravenously sensitizes pancreatic neuroendocrine tumors and metastases to immune checkpoint blockade

Mitsuko Inoue, Minah Kim, Tomoyoshi Inoue, Madeline Tait, Thomas Byrne, Maximilian Nitschké, Patrizia Murer, Howard Cha, Aishwarya Subramanian, Naomi De Silva, Teresa Chiaverotti, and Donald M. McDonald

Table S1. **Flow cytometric analysis of immune cell influx into tumors of RT2;B6 mice**
Values are mean ± SEM of the number of 9 immune cell phenotypes expressed per 50,000 cells analyzed by flow cytometry after isolation from tumors of RT2;B6 mice 5 days after treatment with Vehicle, aPD1, mpJX, or mpJX+aPD1. Values are ranked in decreasing abundance after mpJX+aPD1. NK, natural killer cells; T reg, regulatory T cells; NKT cells, natural killer T cells; M1 and M2, macrophage subtypes. Values for individual mice in each group are shown in Figure 4F. Student's *t*-test: **p* value < 0.05 vs. Vehicle (*n* = 5-6 mice/group).

Phenotype	Sorting strategy after Live/$CD45^+$	Vehicle	aPD1	mpJX	mpJX+aPD1
CD8	$CD19^-/NK1.1^-/TCRB^+/CD8^+/CD4^-$	200 ± 39	1142 ± 30*	3049 ± 178*	6715 ± 1106*
B cells	$CD19^+/NK1.1^-$	177 ± 45	2943 ± 1236*	5364 ± 577*	3769 ± 1074*
CD4	$CD19^-/NK1.1^-/TCRB^+/CD8^-/CD4^+$	126 ± 28	1263 ± 548	2130 ± 221*	2511 ± 578*
M1	$CD19^-/NK1.1^-/TCRB^-/CD11c^-/CD64^+/CD11b^{++}$	290 ± 74	309 ± 65	656 ± 72*	1085 ± 198*
DC	$CD19^-/NK1.1^-/TCRB^-/CD11c^+$	129 ± 42	323 ± 116	850 ± 37*	755 ± 94*
M2	$CD19^-/NK1.1^-/TCRB^-/CD11c^-/CD64^+/CD11b^+$	72 ± 25	58 ± 8.3	276 ± 64*	325 ± 78*
NK cells	$CD19^-/NK1.1^+/TCRB^-$	35 ± 4.3	55 ± 16	92 ± 6.0*	163 ± 23*
T reg	$CD19^-/NK1.1^-/TCRB^+/CD8^-/CD4^+/CD25^+/Foxp3^+$	5.0 ± 2.1	49 ± 30	65 ± 5.9*	102 ± 33*
NKT cells	$CD19^-/NK1.1^+/TCRB^+$	3.2 ± 1.2	3.6 ± 1.1	18.6 ± 7.4	8.3 ± 5.3

Table S2. RT2;AB6F1 mice for analysis of liver metastases
Number and gender of RT2;AB6F1 mice in each group used for analysis of treatment effects on liver metastases. Treatment onset age 15.5 weeks. Metastases were visible on the liver surface of 71% of Vehicle-treated mice at 10 days and 79% at 20 days that were subsequently confirmed to have metastases by microscopic analysis after SV40 T-antigen/DAPI staining. Fewer metastases were visible on the liver surface of mpJX+aPD1-treated mice, which had the smallest metastases. All mice (**Total**) were used for all calculations, except for analyses that included only mice with confirmed metastases (**Subset A**), which excluded mice without identified metastases (**Subset B**). Number of metastases reflects the total metastases identified in mice of each gender in each group. Vaccinia virus mpJX-594 (mpJX) injected i.v. as one dose at age 15.5 weeks; anti-PD-1 antibody (aPD1) injected i.p. every other day over 10 or 20 days; mpJX+aPD1 is the combination of mpJX and aPD1 administered together; IHC staining, immunohistochemical staining.

**Treatment duration 10 days**	**Onset**	**Vehicle**	**aPD1**	**mpJX**	**mpJX +aPD1**
Total number of mice in group (**Total**)	18	14	12	14	14
Number of Male/Female (M/F) mice	12/6	9/5	6/6	8/6	7/7
Number of mice with metastases visible on liver surface	8 (44%)	10 (71%)	10 (83%)	8 (57%)	6 (43%)
Number of mice with liver metastases visible by IHC staining (**Subset A**)	10 (56%)	13 (93%)	11 (92%)	11 (79%)	12 (86%)
Number of mice without liver metastases visible by IHC staining (**Subset B**)	8 (44%)	1 (7%)	1 (8%)	3 (21%)	2 (14%)
Number of metastases analyzed (M/F)	70/20	80/29	50/56	80/30	49/39
Survival from onset to end of 10-day treatment (onset age 15.5 weeks)	100%	87.5%	100%	100%	100%
**Treatment duration 20 days**					
Total number of mice in group (**Total**)	18	14	14	16	17
Number of Male/Female (M/F) mice	12/6	9/5	8/6	9/7	9/8
Number of mice with metastases visible on liver surface	8 (44%)	11 (79%)	8 (57%)	7 (44%)	10 (59%)
Number of mice with liver metastases visible by IHC staining (**Subset A**)	10 (56%)	13 (93%)	11 (79%)	12 (75%)	13 (76%)
Number of mice without liver metastases visible by IHC staining (**Subset B**)	8 (44%)	1 (7%)	3 (21%)	4 (25%)	4 (24%)
Number of metastases analyzed (M/F)	70/20	90/35	70/40	80/40	54/70
Survival from onset to end of 20-day treatment (onset age 15.5 weeks)	100%	57%	73%	86%	91%

Table S3. Treatment effects on metastasis number, size, and burden in RT2;AB6F1 mice: all mice included with or without metastases

Values for the number, size, and burden of liver metastasis in RT2;AB6F1 mice at the beginning of the experiment at age 15.5 weeks (Onset controls) or after 10 or 20 days of treatment. Values calculated from data for all mice with or without metastases in each group. Virus mpJX-594 was administered as one i.v. injection (10^7 pfu) on day 0. Anti-PD-1 antibody was administered by i.p. injection (BioXCell RMP1-14, 100 μg) or control IgG2a (BioXCell BE0089, 100 μg) on day 0 and every other day thereafter. Number of liver metastases is expressed per 100 mm^2 liver sections. Diameter of metastases is expressed in millimeters. Metastatic burden is expressed as total area of metastases (mm^2) per mm^2 of liver sections. Student's *t*-test: $p < 0.05$ vs. *Onset control, #Vehicle, §aPD1, †mpJX.

	Onset control	Vehicle	Anti-PD-1 antibody	mpJX-594	mpJX-594 + anti-PD-1
**Treatment duration 10 days**					
Metastasis number/100 mm^2	16.8 ± 4.9	37.4 ± 9.4*	44.1 ± 8.9*	20.1 ± 3.7§	9 ± 3.2#§†
Metastasis diameter (mm)	0.22 ± 0.08	0.62 ± 0.14*	0.43 ± 0.11	0.26 ± 0.05#	0.12 ± 0.03#§†
Metastasis burden (mm^2/mm^2))	1.2 ± 0.58	4.71 ± 1.36*	2.19 ± 0.85	0.66 ± 0.19#	0.18 ± 0.08*#§†
Number (mice)	18	14	12	14	14
**Treatment duration 20 days**					
Metastasis number/100 mm^2	16.8 ± 4.9	123 ± 15.9*	87 ± 17.6*	43.8 ± 10.9*#§	35.2 ± 6.9*#§
Metastasis diameter (mm)	0.22 ± 0.08	0.79 ± 0.12*	0.67 ± 0.17*	0.41 ± 0.12#	0.21 ± 0.05#§
Metastasis burden (mm^2/mm^2)	1.2 ± 0.58	11.49 ± 1.91*	7.38 ± 1.79*	2.44 ± 1.01#§	0.65 ± 0.16#§
Number (mice)	18	14	14	16	17

Table S4. Treatment effects on metastasis number, size, and burden in RT2;AB6F1 mice: only mice with metastases (mice without metastases excluded)
Values for the number, size, and burden of liver metastasis in RT2;AB6F1 mice at the beginning of the experiment at age 15.5 weeks (Onset controls) or after 10 or 20 days of treatment. Values calculated from data for all mice with metastases in each group. Mice without metastases were excluded from these calculations. Virus mpJX-594 was administered as one i.v. injection (10^7 pfu) on day 0. Anti-PD-1 antibody was administered by i.p. injection (BioXCell RMP1-14, 100 µg) or control IgG2a (BioXCell BE0089, 100 µg) on day 0 and every other day thereafter. Number of liver metastases is expressed per 100 mm^2 liver sections. Diameter of metastases is expressed in millimeters. Metastatic burden is expressed as total area of metastases (mm^2) per mm^2 of liver sections. Student's *t*-test: $p < 0.05$ vs. *Onset control, $^{\#}$Vehicle, §aPD1, †mpJX.

	Onset control	Vehicle	Anti-PD-1 antibody	mpJX-594	mpJX-594 + anti-PD-1
**Treatment duration 10 days**					
Metastasis number/100 mm^2	30.2 ± 6.1	40.3 ± 9.6	48.2 ± 8.7	25.6 ± 2.9§	10.5 ± 3.6$^{*\#\S\dagger}$
Metastasis diameter (mm)	0.40 ± 0.12	0.66 ± 0.15	0.47 ± 0.12	0.33 ± 0.04	0.13 ± 0.03$^{*\#\S\dagger}$
Metastasis burden (mm^2/mm^2)$^{)}$	2.17 ± 0.96	5.07 ± 1.41	2.39 ± 0.9	0.84 ± 0.22$^{\#}$	0.21 ± 0.09$^{*\#\S\dagger}$
Number (mice)	10	13	11	11	12
**Treatment duration 20 days**					
Metastasis number/100 mm^2	30.2 ± 6.1	133 ± 13.7*	111 ± 15.8*	58.4 ± 11.8$^{\#\S}$	46.0 ± 6.4$^{\#\S}$
Metastasis diameter (mm)	0.40 ± 0.12	0.85 ± 0.11*	0.85 ± 0.18	0.54 ± 0.13	0.27 ± 0.05$^{\#\S}$
Metastasis burden (mm^2/mm^2)	2.17 ± 0.96	12.38 ± 1.8*	9.39 ± 1.85*	3.25 ± 1.28$^{\#\S}$	0.85 ± 0.18$^{\#\S}$
Number (mice)	10	13	11	12	13

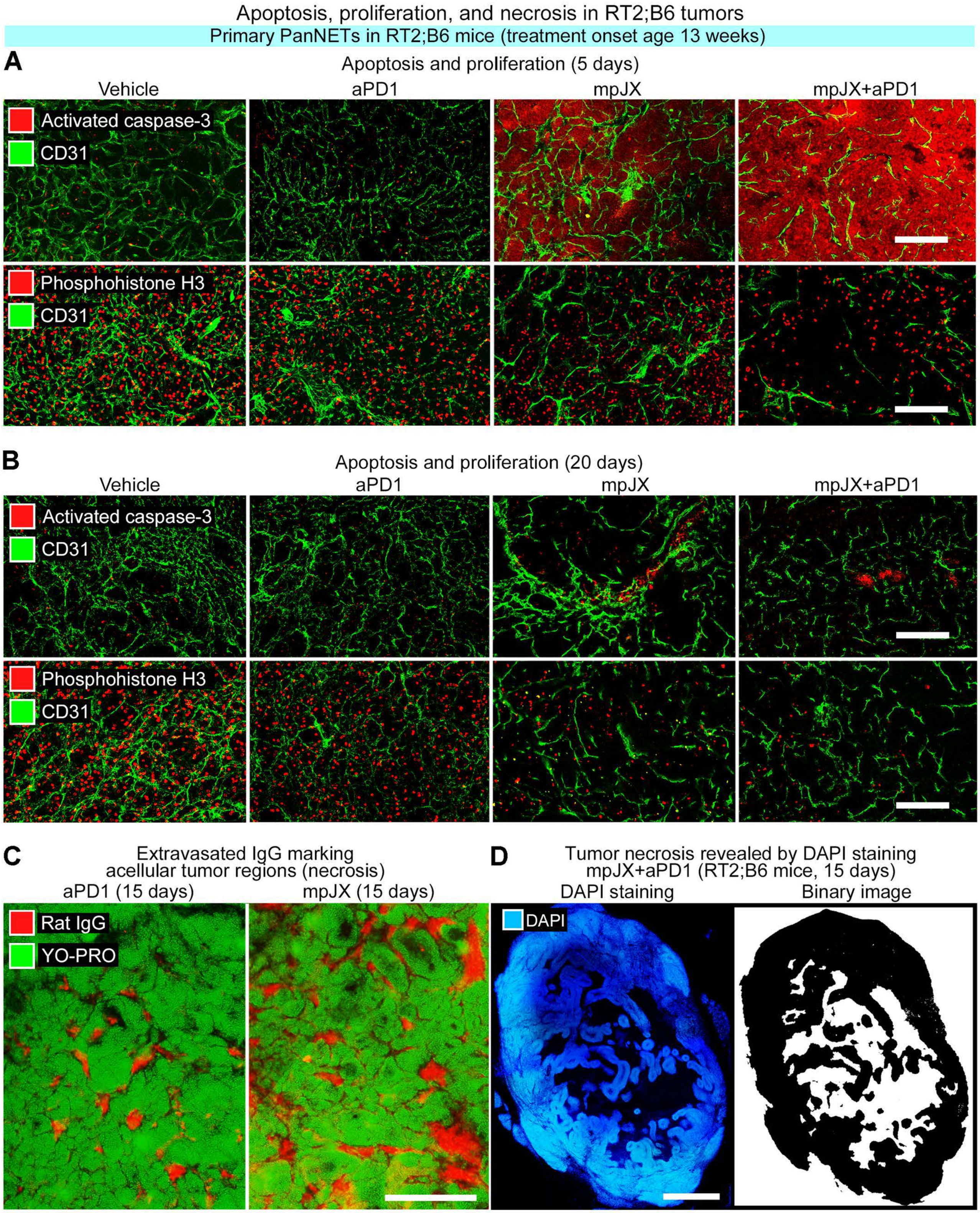

Figure S1. Apoptosis, proliferation, and necrosis in RT2;B6 tumors
(A and B) Confocal microscopic images comparing amount and distribution of apoptosis (upper rows: activated caspase-3, red), proliferation (lower rows: phosphohistone H3, red), and blood vessels (CD31, green) in tumors at 5 days in A and 20 days in B after Vehicle, aPD1, mpJX, or mpJX+aPD1. Apoptosis is greatest at 5 days after mpJX+aPD1 (A, upper right), and suppression of proliferation is greatest at 20 days after mpJX+aPD1 (B, lower right). Scale bar, 200 µm. (C) Fluorescence microscopic images of cell nuclei (YO-PRO-1, green) and extravasated rat IgG (Cy3 anti-rat IgG, red) in tumors at 15 days after aPD1 (left) or mpJX (right), for comparison to similar images after Vehicle or mpJX+aPD1 in Figure 2A. Red patches of extravasated normal rat IgG2a coincide with regions of necrosis marked by absence of YO-PRO-1 staining of cell nuclei. Scale bar, 400 µm. (D) Fluorescence microscopic image of DAPI^{+} viable cells (left, blue) and unstained regions (black) in RT2;B6 tumor at 15 days after mpJX+aPD1 and the same image converted into a binary image (right) in Photoshop for measuring the proportion of white pixels (necrosis) with ImageJ. Scale bar, 1 mm.

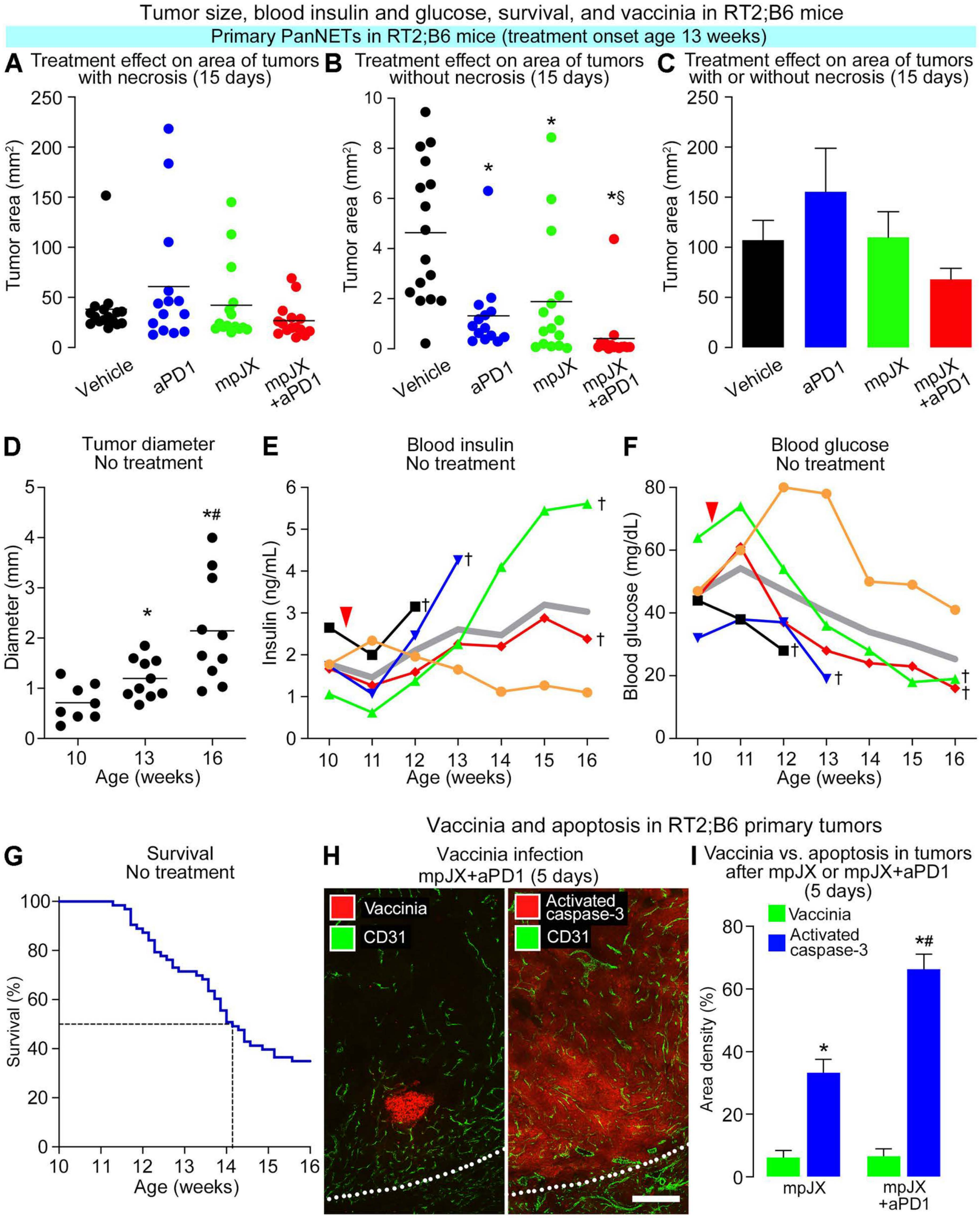

Figure S2. Tumor size, blood insulin and glucose, and survival in RT2;B6 mice
(A-C) Dot plot and bar graph showing the mean area (mm^2) of tumors with necrosis in A, tumors without necrosis in B, and tumors with or without necrosis in C in each mouse at 15 days after treatment. Student's *t*-test: $p < 0.05$ compared to *Vehicle, #mpJX. n = 14-16 mice/group. (D) Dot plot showing the rate of tumor growth in untreated RT2;B6 mice indicated by mean tumor diameter at 10 weeks (0.7 mm), 13 weeks (1.2 mm), and 16 weeks (2.1 mm). Student's *t*-test: $P < 0.05$ compared to *10 weeks or #13 weeks. n = 8-10 mice/group. (E and F) Line plots showing changes in blood insulin (E, ng/mL) and glucose (F, mg/dL) in untreated RT2;B6 mice from age 10 to 16 weeks. Values for 5 individual mice are shown as black, blue, green, red, and orange curves, and mean is shown by the thick gray line. Red triangles mark when sucrose was added to diet at age 10 weeks after taking the initial blood sample. Crosses mark the death of 4 mice. Insulin increased and glucose decreased over time. Blood insulin above 2 ng/mL or blood glucose under 30 mg/dL after age 12 weeks indicated high risk of death. (G) Kaplan-Meier curve showing decreasing survival of untreated RT2;B6 mice from age 10 to 16 weeks (n = 63 mice). Dotted line indicates 50% survival at 14.1 weeks. Only 34.9% of the mice survived to age 16 weeks. (H) Confocal images of RT2;B6 tumors 5 days after mpJX+aPD1 showing focal patches of vaccinia antigen (left, red) and widespread staining for activated caspase-3 (right, red). Tumor vessels (CD31, green). (I) Bar graph comparing the limited amount of vaccinia infection (vaccinia antigen) to the widespread apoptosis (activated caspase-3 staining) in RT2;B6 tumors at 5 days after mpJX and even greater difference after mpJX+aPD1 due to more extensive apoptosis. ANOVA: $p < 0.05$ compared to *Vaccinia, #mpJX. n = 5-8 mice/group.

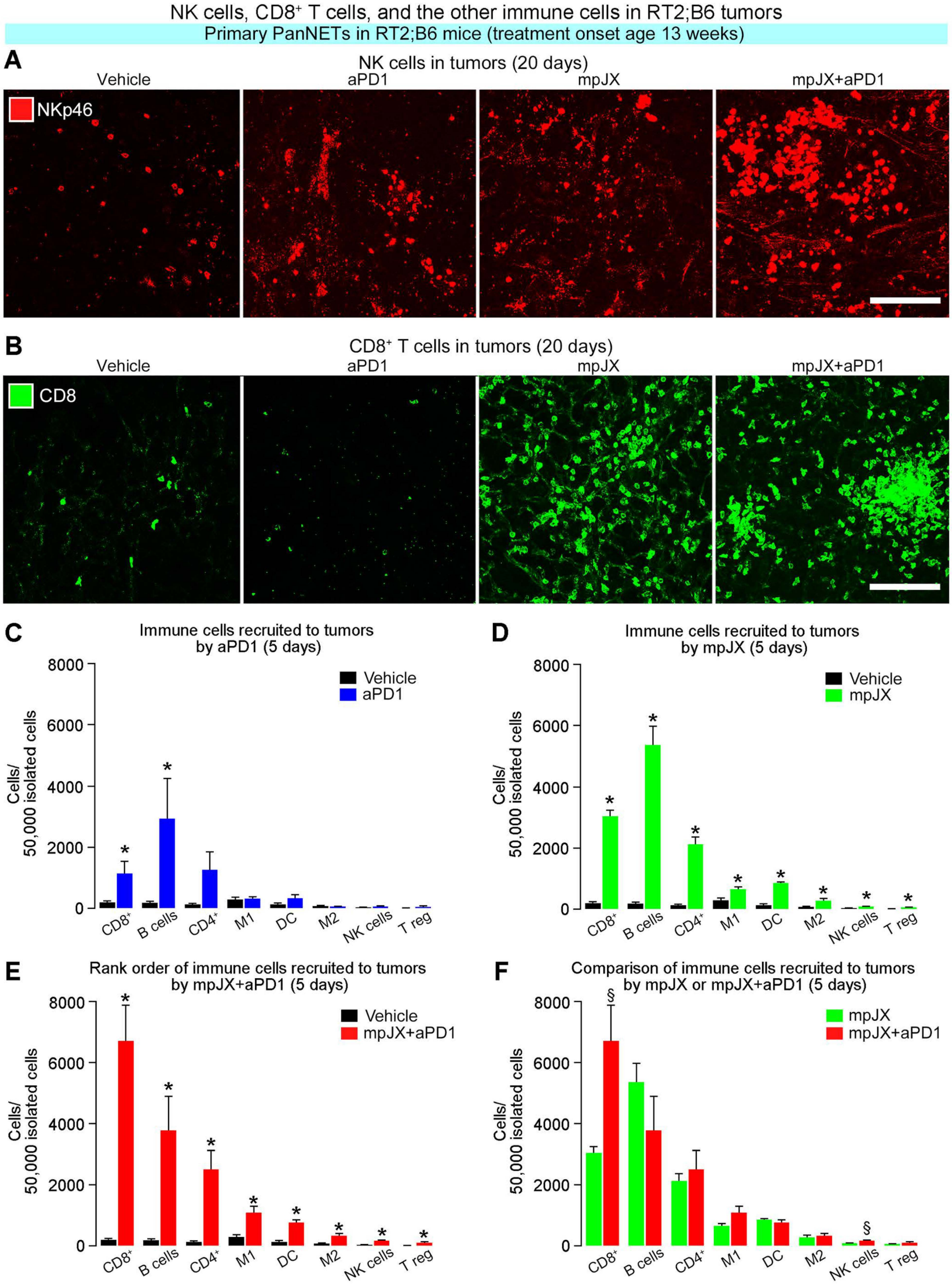

Figure S3. **Treatment-related changes in immune cell influx into RT2;B6 tumors**
(A and B) Confocal microscopic images of NK cells (NKp46, red) and CD8+ T cells (CD8, green) at 20 days after Vehicle, aPD1, mpJX, or mpJX+aPD1 showing persistent increase in NKp46+ cells after aPD1, mpJX, or mpJX+aPD1 and CD8+ cells after mpJX or mpJX+aPD1. Scale bar, 200 μm. Corresponding measurements of NKp46+ cells and CD8+ cells are shown in Figures 3D and 3F. (C-F) Bar graphs of flow cytometric data comparing treatment effects on rank order of number of immune cells isolated from tumors of RT2;B6 mice after treatment over 5 days. Sorting strategy and original data are in Figure 4F and Table S1. Values are expressed as number of cells per 50,000 isolated cells. Mean ± SEM. Student's *t*-test: $p < 0.05$ compared to *Vehicle or §mpJX. n = 5-6 mice/group.

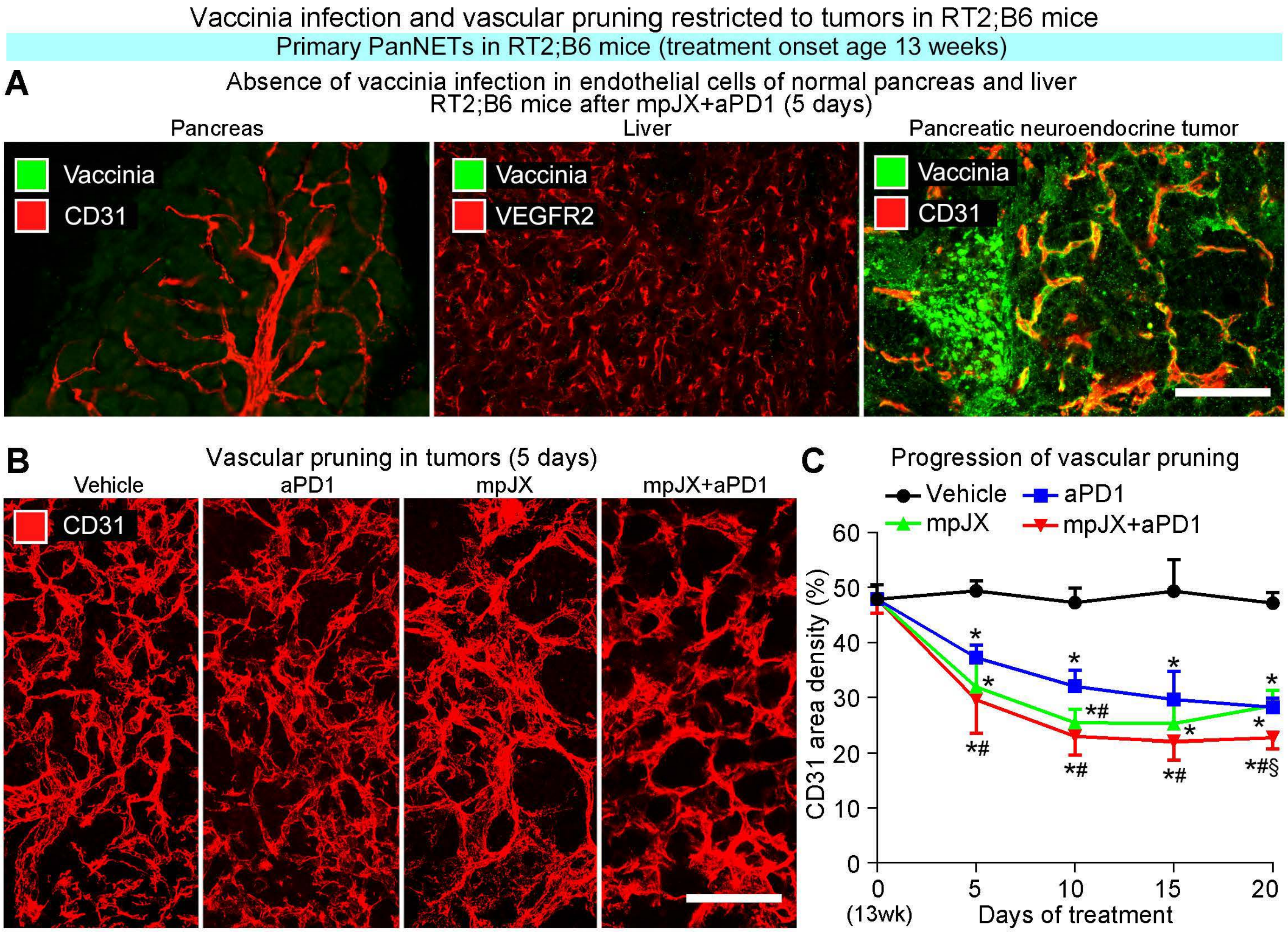

Figure S4. **Treatment-related changes in vasculature of tumors and absence of vaccinia infection of normal organs of RT2;B6 mice**

(A) Confocal microscopic images of pancreatic blood vessels (CD31, red) and liver sinusoids (VEGFR2, red) showing no vaccinia infection (vaccinia antigen, green) in normal pancreas acini or liver sinusoids but strong infection in primary pancreatic islet tumor at 5 days after mpJX+aPD1. (B) Confocal microscopic images showing differences in RT2;B6 tumor vasculature (CD31, red) at 5 days after Vehicle, aPD1, mpJX, or mpJX+aPD1. Scale bar, 100 µm. (C) Line plots showing stable vascular density in RT2;B6 tumors over 20 days of treatment with Vehicle compared to progressive vascular pruning after aPD1, mpJX, or mpJX+aPD1. Related measurements are shown in Figures 5D. Tumor vascularity decreased more rapidly after mpJX or mpJX+aPD1 than after aPD1, but the reduction at 20 days was the same after mpJX or aPD1 and was significantly greater after mpJX+aPD1. ANOVA: $p < 0.05$ compared to *Vehicle, $^{\#}$aPD1, §mpJX. n = 5-12 mice/group.

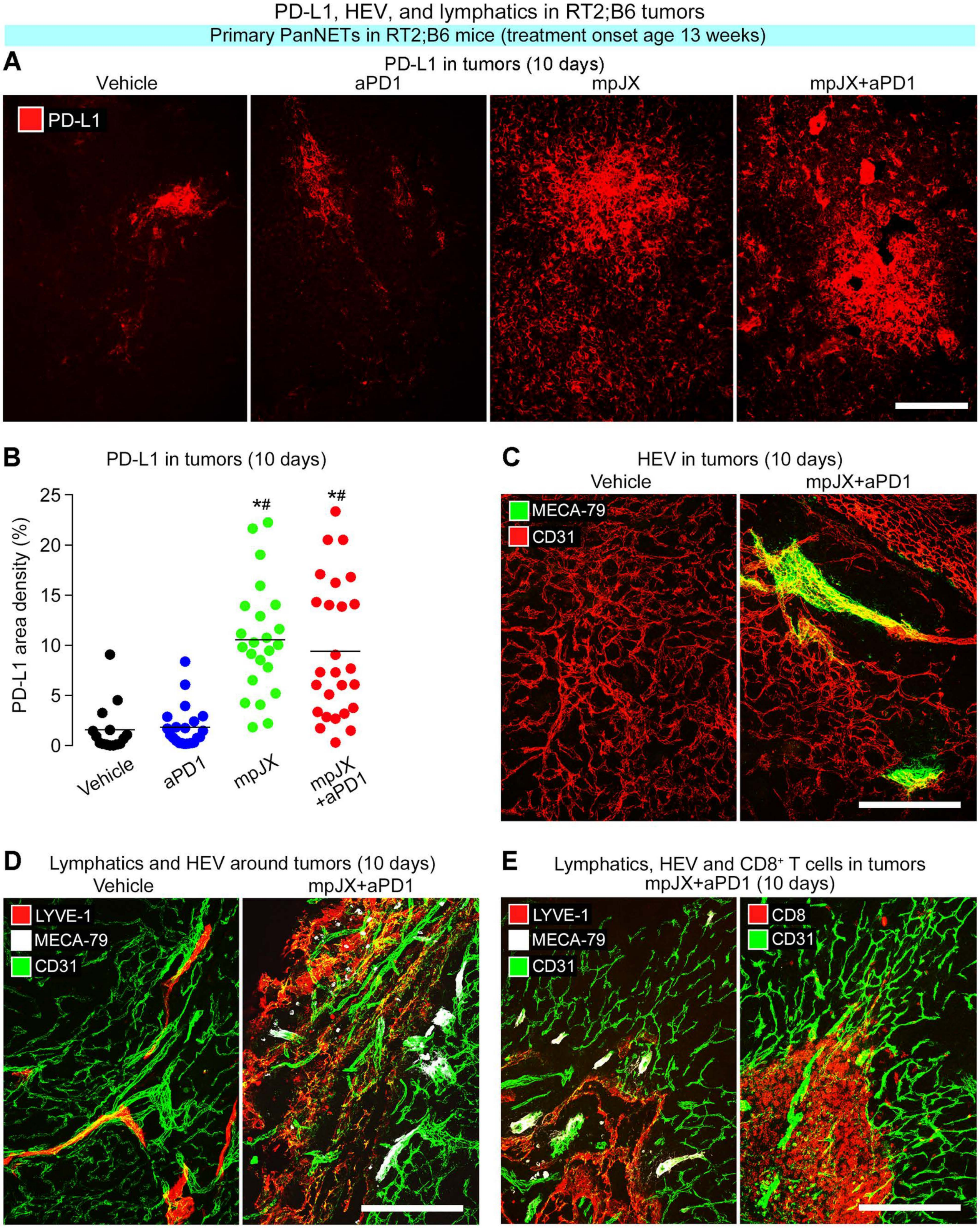

Figure S5. Treatment-related changes in PD-L1, HEV, and lymphatics in RT2;B6 tumors
(A) Confocal microscopic images of PD-L1 immunoreactivity (red) in tumors showing large increases after mpJX or mpJX+aPD1 but no difference between Vehicle and aPD1 over 10 days. Scale bar, 100 µm. (B) Measurements confirming the increase in PD-L1 staining in tumors after mpJX or mpJX+aPD1 but not after aPD1 over 10 days. The scatter in the dots shows that the increase in PD-L1 staining after mpJX or mpJX+aPD1 was variable from tumor to tumor. ANOVA: $p < 0.05$ compared to *Vehicle, $^{\#}$aPD1. n = 15-26 tumors/group. (C) Confocal microscopic images showing the absence of high endothelial venules (HEV, MECA-79, green) in a tumor at 10 days after Vehicle (left panel) and the presence of HEV after mpJX+aPD1 (right panel). Blood vessels (CD31, red). Scale bar, 200 µm. (D and E) Confocal microscopic images of tumors with few or no peritumoral lymphatics (LYVE-1, red) and HEV (MECA-79, white) at 10 days after Vehicle (D, left panel) compared to abundant lymphatics and HEV after mpJX+aPD1 (D right panel, E, both panels). Blood vessels (CD31, green). Some lymphatics and HEV (E, left panel) were located in a region with abundant CD8^{+} cells shown in an adjacent section (E, right panel, red). Scale bar, 200 µm.

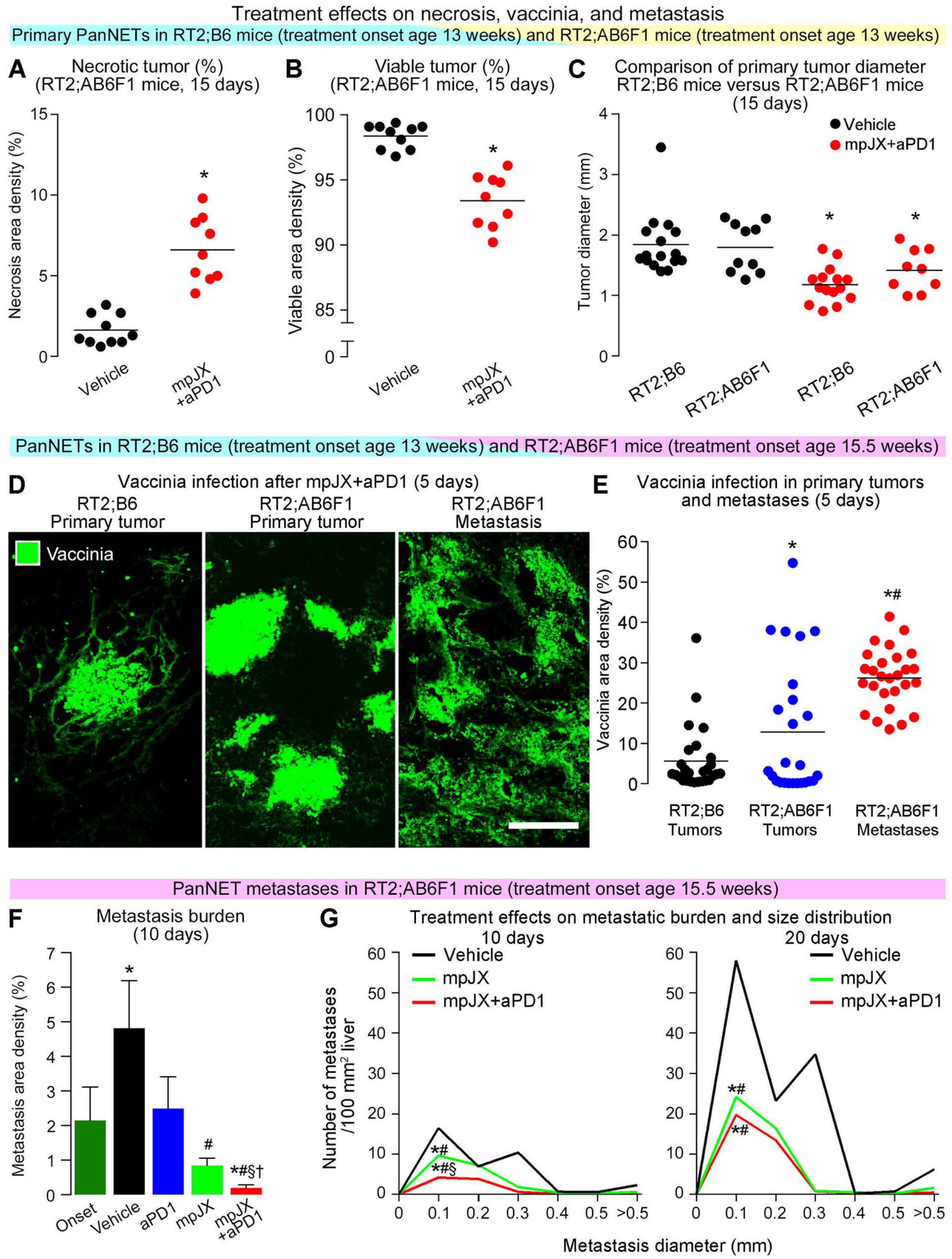

Figure S6. Treatment effects on necrosis, vaccinia and metastasis

(A and B) Amount of necrosis in primary tumors in RT2;AB6F1 mice was greater and amount of viable tumor was less at 15 days after mpJX+aPD1 than after vehicle. Each dot is mean value for one mouse. (C) Primary tumor diameter was smaller at 15 days after mpJX+aPD1 than after vehicle both in RT2;B6 mice and in RT2;AB6F1 mice. (A-C) Student's *t*-test: $p < 0.05$ compared to *Vehicle. n = 9-10 mice/group in A and B or 9-16 mice/group in C. (D and E) Confocal microscopic images and dot plots showing that vaccinia antigen staining (green) was more widespread in primary tumors (13%) and metastases (27%) in RT2;AB6F1 mice than in primary tumors in RT2;B6 mice (6%). Student's *t*-test: $p < 0.05$ compared to *RT2;B6 primary tumors, #RT2;AB6F1 primary tumors. n = 25-27 primary tumors or metastases/group. Scale bar, 200 µm. (F) Metastatic burden in RT2;AB6F1 mice with liver metastases (mice lacking metastases excluded) showing significantly smaller values after mpJX+aPD1 than at the Onset, indicative of regression. Other treatment-related differences were similar to those found when mice with and mice without metastases were considered together (see Figure 8F). Student's *t*-test: $p < 0.05$ compared to *Onset, #Vehicle, §aPD1, †mpJX. n = 10-13 mice/group. (G) Treatment effects on the size distribution of metastases in the liver of RT2;AB6F1 mice (mice lacking metastases excluded) showing significantly smaller metastases after mpJX or mpJX+aPD1 than after Vehicle at 10 days (left) and 20 days (right) after the onset of treatment. At 10 days, metastases were smaller after mpJX+aPD1 than after mpJX. Kolmogorov-Smirnov two-sample test: $p < 0.001$ compared to *Vehicle. Student's *t*-test: $p < 0.05$ compared to #Vehicle, §mpJX. n = 10-13 mice/group.

Gender differences in metastasis number, size, and burden in RT2;AB6F1 mice

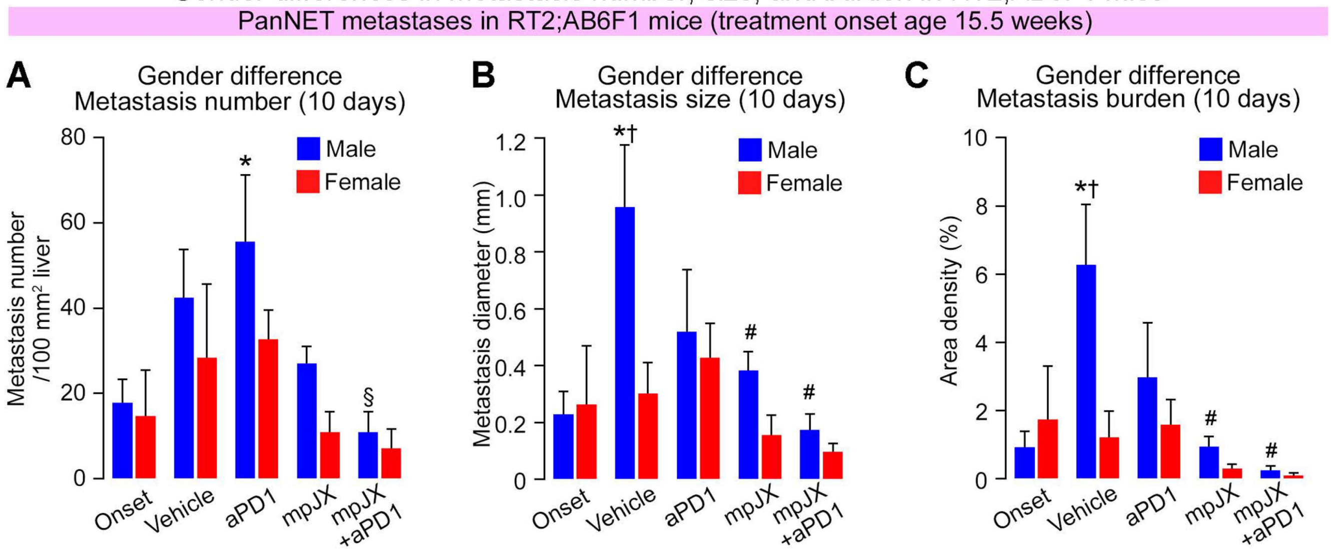

Figure S7. Gender differences in metastasis number, size, and burden in RT2;AB6F1 mice
(A-C) Male/female comparison of values for metastasis number/100 mm^2 liver in A, diameter (mm) in B, and metastatic burden (area density, %) in C at 10 days after the onset of treatment showing significantly greater metastatic burden in males than females after Vehicle. ANOVA: $p < 0.05$ compared to †Females. In males, metastasis size and area density were significantly smaller at 10 days after mpJX or mpJX+aPD1 than Vehicle, and were larger after Vehicle than at the Onset. ANOVA, $p < 0.05$ compared to *Onset, #Vehicle, §aPD1. Despite similar patterns in females, differences were not significant because metastases were smaller and group size was not powered for gender analysis. n = 6-12 males and 5-7 females/group; mice with or without metastases included (see Table S2).

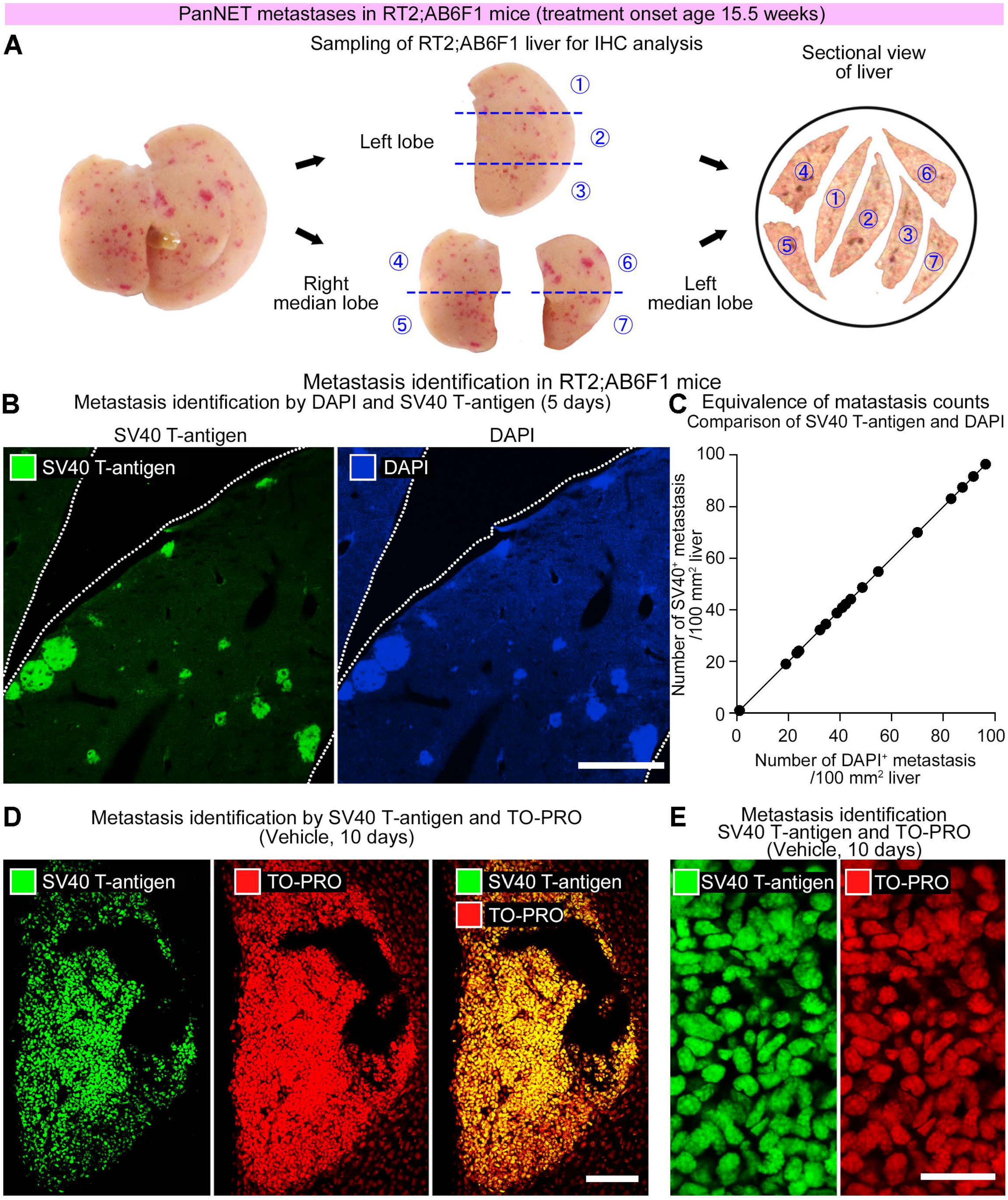

Figure S8. Approach used to measure metastases in RT2;AB6F1 mice
(A) Approach for sampling liver for morphometric analysis of metastases in RT2;AB6F1 mice and identifying metastases by immunohistochemical (IHC) and DAPI staining. After perfusion fixation, liver (right lobe and caudate lobe were removed) was divided into 7 pieces (left lobe 3 pieces, left median lobe 2 pieces, right median lobe 2 pieces), embedded in OCT, and sectioned. (B) Fluorescence microscopic images of liver of RT2;AB6F1 mouse at 5 days after Vehicle confirming that the same metastases can be identified by staining for SV40 T-antigen (left, green) or DAPI (right, blue). Scale bar,1 mm. (C) Linear regression showing identical counts of metastases in liver sections stained for SV40 T-antigen and DAPI. n = 18 mice. (D) Confocal microscopic images of liver metastasis stained for SV40 T-antigen (left, green) and TO-PRO-1 (center, red) with the merged image (right, yellow) at 10 days after Vehicle. Scale bar,1 mm. (E) Higher magnification views of left and center images in D Scale bar, 25 μm.

Supplemental Methods

Tumor models

RIP1-Tag2 transgenic mice in the C57BL/6 background (RT2;B6 mice), which develop spontaneous pancreatic islet cell carcinomas,[1] were bred by mating RT2;B6 males to wild-type C57BL/6 females (The Jackson laboratory, Bar Harbor, ME). Hybrid RT2;AB6F1 mice[2] were generated by mating RT2;B6 males to wild-type A/J females (The Jackson Laboratory). SV40 T-antigen transgene-positive mice were identified by polymerase chain reaction (PCR) genotyping of tail-tip DNA. RT2;B6 and RT2;AB6F1 mice received a diet with added 50% sucrose from the age of 10 weeks to ameliorate hypoglycemia.

Virus preparation and iv injection

On the day 0 of experiments, mpJX virus stock at 1×10^9 pfu/mL stored at -80°C was thawed at 4°C for 1 hr and immediately put on ice.[3] Each aliquot of mpJX was dispersed by pulse vortex for 30 sec, diluted to 1×10^8 pfu/mL with PBS, and vortexed again for 30 sec in a BSL2 hood. Doses of 1×10^7 pfu were withdrawn into 100 µL syringes, stored on ice until injection within 2 hr, and vortexed for 30 sec just before injection.

Blood glucose and serum insulin measurements

Blood glucose was measured in blood drawn from a tail vein without anesthesia from RT2;B6 mice after 3-hr fasting with free access to water in the morning of day 0 and again at the end of treatment (ACCU-CHEK Performa, Roche). Blood glucose was also measured in 3 untreated male and 2 female control RT2;B6 mice weekly from age 10 to 16 weeks. Serum insulin was measured in another sample drawn from a tail vein of treated and control mice and stored in BD Microtainer tubes (Gold). Tubes were inverted 5x to promote coagulation over 30 min at room temperature. Fully clotted serum was removed and frozen after centrifugation at

7000 rpm for 10 min. Insulin was measured with the Ultra-Sensitive Mouse Insulin ELISA Kit (Crystal Chem USA, Elk Grove Village, IL), according to the manufacturer's instructions.

Tissue processing for immunohistochemistry

After fixation by perfusion of 1% paraformaldehyde (PFA) through the left cardiac ventricle, the pancreas and liver were removed and fixed with 1% PFA at 4°C for 1 hour. After washing twice every 10 min with PBS, specimens were immersed in 30% sucrose at 4°C overnight, embedded in OCT (Tissue-Tek® O.C.T. Compound, SAKURA), and frozen. Cryostat sections 80 μm in thickness of pancreas or liver were stained by immunohistochemistry using combinations with 2 or 3 primary antibodies listed below.

Antibodies for immunohistochemistry and fluorescent stains for cell identification

Target	Vendor	Catalog No.	Species	Dilution
Activated caspase-3	Cell Signaling	9579	Rabbit	1:1000
CD8	UCSF Core	-	Rat	1:500
CD4	Bio-Rad	MCA4635	Rat	1:500
CD19	Cell Signaling	D4V4B	Rabbit	1:500
CD31	Invitrogen	MA3105	Armenian Hamster	1:500
Desmin	Millipore	04-585	Rabbit	1:500
LYVE-1	AngioBio Co	11-034	Rabbit	1:500
MECA-79	BD biosciences	553863	Wistar Furth Rat	1:500
NKp46	R&D systems	AF2225	Goat	1:250
Phosphohistone H3	Millipore	06-570	Rabbit	1:500
Pimonidazole	Chemicon	HP2-100 Kit	-	1:100
SV40 T-antigen	Santa Cruz	SC-20800	Rabbit	1:500
S100A8	R&D systems	AF3059	Goat	1:500
TO-PRO-1	Invitrogen	T3602	-	1:3000
Vaccinia	Quartett	VA003	Rabbit	1:1000
VEGFR2	R&D systems	AF644	Goat	1:500
YO-PRO-1	Invitrogen	Y3603	-	1:3000

Secondary antibodies were Cy3, Alexa-488, or Alexa-647-labeled donkey anti-goat, donkey/goat anti-rabbit, donkey/goat anti-rat, or donkey/goat anti-Armenian hamster IgG or IgM antibody (Jackson ImmunoResearch; all diluted 1:500). Regions of hypoxia in tumors were identified with pimonidazole (1.5 mg/mouse in 100 µL of PBS, Hypoxyprobe Plus Kit HP2; Chemicon) injected i.p. 1 hr before the perfusion. Pimonidazole adducts in tissue was stained with FITC-conjugated mouse anti-pimonidazole hydrochloride (1:100, Chemicon). PD-L1 staining used tyramide signal amplification (SAT704A001EA, PerkinElmer) with horseradish peroxidase-labeled goat anti-rat IgG (1:1000; eBioscience).[4] Extravasated rat IgG2a was localized in tumors with Cy3-labeled anti-rat IgG. Cell nuclei were stained with TO-PRO-1, YO-PRO-1, or Vectashield mounting medium containing DAPI (Vector Laboratories).

Measurement of apoptosis, proliferation, vaccinia, vasculature, hypoxia, and PD-L1 in primary tumors

Fractional area of tumor cell apoptosis (activated caspase-3) and proliferation (phosphohistone H3) in 80-µm sections of the pancreas were measured in fluorescence microscopic images of the 5 largest tumors captured with an Olympus DP73 camera on a Zeiss Axiophot fluorescence microscope (2.5x objective and 1x Optovar). Large tumors requiring multiple images to include the entire tumor were montaged into single images in Photoshop (version 21.1.3). Staining for vaccinia, CD31, pimonidazole, or PD-L1 was similarly measured in fluorescence microscopic images of the 5 largest tumors (10x objective, 1x Optovar, image area = 1.36 mm^2, 1200 pixels x 1600 pixels). One region was sampled per tumor when the immunoreactivity was evenly distributed. Two regions were sampled in tumors when the distribution was uneven. The number of pixels with immunofluorescence above a predetermined intensity threshold was measured with ImageJ (version 1.52s, http://imagej.nih.gov/ij/)[5] and expressed as the percent of total pixels (area density, %).

Measurement of primary tumor size

Tumor size was measured in fluorescence microscopic images of the 10 largest tumors in an 80-μm section of pancreas from each mouse (5x objective, 1x Optovar). Large tumors requiring multiple images were montaged into single images in Photoshop. When fewer than 10 tumors were present, all tumors were measured. Tumor sectional area was measured by tracing the perimeter in ImageJ and expressed as tumor area (mm^2) and diameter (mm) calculated from the area assuming circularity.

Measurement of necrosis and viable tumor

The amount of necrosis was measured in fluorescence microscopic images (5x objective, 1x Optovar) of the 5 largest primary tumors in each mouse. Large tumors requiring multiple images were montaged into single images in Photoshop. Total sectional area of tumors stained for SV40 T-antigen or DAPI was measured with ImageJ. Necrotic regions, identified by absence of DAPI staining, were measured in a binary image made from the original image in Photoshop (Figure S1D) and expressed as percent of total tumor sectional area. The amount of viable tumor was calculated as the total tumor area minus the area of necrosis and expressed as percent of total tumor area.

Measurement of $CD8^+$ T cells and NK cells in primary tumors

Cells stained for CD8 or NKp46 were counted in confocal microscopic images prepared as 2-dimensional projections of a 10-image Z-stack (optical slice = 1.54 μm). The region of greatest $CD8^+$ cell or $NKp46^+$ cell abundance was measured in 80-μm sections of each of the 5 largest tumors in each mouse (20x objective, 1x zoom, image area = 0.2 mm^2). $CD8^+$ cells and $NKp46^+$ cells were counted with the cell counter function of ImageJ and expressed as the mean number of cells per square millimeter of tumor sections in each mouse.

Measurement of immune cells around necrotic regions

$CD8^+$ cells, $NKp46^+$ cells, $CD4^+$ cells, $CD19^+$ cells (B cells), and $S100A8^+$ cells (neutrophils) in regions bordering necrosis in 80-μm sections of the 5 largest primary tumors were counted in confocal microscopic images (20x objective, 2x zoom, image area = 0.05 mm^2). The perimeter of necrotic regions was measured with ImageJ. The abundance of each cell type was expressed as the mean numerical density (cells/mm length of perimeter of necrotic region) for each mouse.

Measurement of metastasis size and number

Metastases identified as clusters of SV40 T-antigen$^+$ cells collectively measuring 50 μm or larger in diameter were analyzed in 80-μm thick sections of 7 regions of liver of RT2;AB6F1 mice (Figures S8A and S8B, left). When SV40 T-antigen staining was weak, metastases were identified as densely packed DAPI-stained nuclei (Figure S8B, right), which gave identical values (Figure S8C). Metastases were counted in liver sections (10x objective, 1x Optovar) and expressed as the number per 100 square millimeters of liver sections in each mouse. The area of the 10 largest metastases in liver sections from each mouse was measured in fluorescence microscopic images (10x objective, 1x Optovar) by tracing the perimeter with ImageJ. The area of metastases was converted to square millimeters, and the diameter (millimeters) was calculated from the area assuming circularity. When fewer than 10 metastases were present, all metastases were measured. Otherwise, the size of the remaining population of metastases was sampled by measuring 20 additional metastases across the size range. Metastasis burden was calculated as the mean area of < 30 metastases measured (mm^2) times the number of metastases per square millimeter of liver sections (number/mm^2). Values for males and females were analyzed separately.

Measurement of vaccinia, apoptosis, proliferation, and vasculature in metastases

Fractional areas (area density, %) of staining for vaccinia, activated caspase-3, phosphohistone H3, and CD31 in fluorescence microscopic images of the 5 largest metastases in 80-µm sections of liver (10x objective, 1x Optovar) of RT2;AB6F1 mice were measured by the same methods as for primary tumors.

Measurement of HEV around primary tumors in RT2;AB6F1 mice

HEV around the 5 largest primary tumors in RT2;AB6F1 mice at 10 or 20 days after the onset of treatment were counted in sections stained for MECA-79 and CD31. Tumor circumference was measured in fluorescence microscopic images (2.5x objective, 1x Optovar) by tracing the perimeter with ImageJ. The abundance of HEV visible by confocal microscopy was expressed as the mean numerical density (HEV/centimeter tumor circumference) for the 5 tumors in each mouse.

Measurement of CD8+ T cells and NK cells in metastases

Cells stained for CD8 or NKp46 were counted in confocal microscopic images (20x objective, 1x zoom, image area = 0.2 mm^2) of the region of greatest abundance in 80-µm sections of each of the 5 largest liver metastases in each mouse, by the same approach used for counting these cells in primary tumors. Metastasis area was measured in the same images by tracing the perimeter with ImageJ and expressed in square millimeters. The abundance of $CD8^+$ cells and $NKp46^+$ cells was expressed as the mean number of cells per square millimeter of metastasis in each mouse.

Depletion of $CD8^+$ cells and NK cells in RT2;B6 mice

In $CD8^+$-cell depletion studies, mice received one i.p. injection of 400 µg of anti-CD8 antibody (BioXCell, BE0061, Clone 2.43) or normal rat IgG2a (BioXCell BE0089, West

Lebanon, NH) in 100 µL on days -2, -1, 1, and 3, 5, 7, and 9. In NK-cell depletion studies, mice received one i.p. injection of 100 µg of anti-NK1.1 antibody (BioXCell, BE0036) or normal rat IgG2a in 100 µL on days -1, and 5. On day 0, mice received mpJX or Vehicle by i.v. injection and aPD1 or normal IgG2a by i.p. injection 6 hr after the virus and every other day until the mice were studied on day 10.

The extent of depletion of $CD8^+$ cells or NK cells was assessed by flow cytometry in peripheral blood withdrawn by cardiac puncture and transferred to a tube coated with EDTA containing 1-2 µL heparin. Plasma was discarded and erythrocytes in the pellet were lysed. Samples were stained with antibodies to CD45, CD3e, CD4, and CD8 for assessing $CD8^+$-cell depletion or with antibodies to CD45, CD11b, CD3, and NK1.1 for assessing NK-cell depletion (see list below). Samples were analyzed by flow cytometry (BD LSR Fortessa™) with FlowJo software (8.8.6 and X).

Antibodies used to assess depletion of $CD8^+$ cells and NK cells by flow cytometry

**$CD8^+$ cell depletion**	**Vender**	**Catalog number**	**Dilution**
Anti-mouse CD45, APC-Cy7	BioLegend	103115	1:300
Anti-mouse CD3e, FITC	BioLegend	152303	1:300
Anti-mouse CD4, Alexa Fluor 647	BioLegend	100426	1:300
Anti-mouse CD8, PerCP	BD	561109	1:300

**NK cell depletion**	**Vender**	**Catalog number**	**Dilution**
Anti-mouse CD45, AF700	BioLegend	103127	1:100
Anti-mouse CD3, PerCP	BioLegend	100325	1:100
Anti-mouse NK1.1, APC	BioLegend	108709	1:100
Anti-mouse CD11b, Buv395	BD	742643	1:100

Flow cytometric analysis of immune cells in RT2;B6 mouse tumors

Mice treated with Vehicle, aPD1, mpJX, or mpJX+aPD1 over 5 days were perfused through the left ventricle with cold PBS for approximately 1 min, and the pancreas was

removed. Tumors were isolated from the exocrine pancreas under a dissecting microscope. After tumor digestion in collagenase II and IV solution (625 U/mL, Gibco) with DNase (60 U/mL, Roche), erythrocytes were lysed, and dissociated cells were stained with the antibodies listed below and permeabilized overnight. The following day, cells were stained with anti-mouse Foxp3 and analyzed by flow cytometry (BD LSR Fortessa™) with FlowJo software (8.8.6 and X).

Antibodies used to assess immune cell influx into tumors by flow cytometry

	Vender	Catalog number	Dilution
Anti-mouse CD45, AF700	BioLegend	103127	1:100
Anti-mouse CD19, PE-CF594	BioLegend	115554	1:100
Anti-mouse TCRB, Bv510	BioLegend	109233	1:100
Anti-mouse CD4, BB700	BD	566408	1:200
Anti-mouse CD8, Bv786	BioLegend	100749	1:100
Anti-mouse CD25, PE	BioLegend	101903	1:100
Anti-mouse NK1.1, APC	BioLegend	108709	1:100
Anti-mouse CD11b, Buv395	BD	742643	1:100
Anti-mouse CD11c, Bv711	BD	563048	1:100
Anti-mouse CD64, PE-Cy7	BioLegend	139313	1:100
Anti-mouse Foxp3, AF488	BioLegend	126405	1:100

References

1. Hanahan, D. (1985). Heritable formation of pancreatic beta-cell tumours in transgenic mice expressing recombinant insulin/simian virus 40 oncogenes. Nature *315*, 115-122.
2. Kobayashi, S., Contractor, T., Vosburgh, E., Du, Y.N., Tang, L.H., Clausen, R., and Harris, C.R. (2019). Alleles of Insm1 determine whether RIP1-Tag2 mice produce insulinomas or nonfunctioning pancreatic neuroendocrine tumors. Oncogenesis *8*, 16.
3. Kim, M., Nitschke, M., Sennino, B., Murer, P., Schriver, B.J., Bell, A., Subramanian, A., McDonald, C.E., Wang, J., Cha, H., et al. (2018). Amplification of Oncolytic Vaccinia Virus Widespread Tumor Cell Killing by Sunitinib through Multiple Mechanisms. Cancer Res *78*, 922-937.
4. Chon, H.J., Lee, W.S., Yang, H., Kong, S.J., Lee, N.K., Moon, E.S., Choi, J., Han, E.C., Kim, J.H., Ahn, J.B., et al. (2019). Tumor Microenvironment Remodeling by Intratumoral Oncolytic Vaccinia Virus Enhances the Efficacy of Immune-Checkpoint Blockade. Clin Cancer Res *25*, 1612-1623.

5. Inai, T., Mancuso, M., Hashizume, H., Baffert, F., Haskell, A., Baluk, P., Hu-Lowe, D.D., Shalinsky, D.R., Thurston, G., Yancopoulos, G.D., et al. (2004). Inhibition of vascular endothelial growth factor (VEGF) signaling in cancer causes loss of endothelial fenestrations, regression of tumor vessels, and appearance of basement membrane ghosts. Am J Pathol *165*, 35-52.
